# The Search for New Anticonvulsants in a Group of (2,5-Dioxopyrrolidin-1-yl)(phenyl)Acetamides with Hybrid Structure—Synthesis and In Vivo/In Vitro Studies

**DOI:** 10.3390/ijms21228780

**Published:** 2020-11-20

**Authors:** Michał Abram, Marcin Jakubiec, Anna Rapacz, Szczepan Mogilski, Gniewomir Latacz, Rafał M. Kamiński, Krzysztof Kamiński

**Affiliations:** 1 Department of Medicinal Chemistry, Faculty of Pharmacy, Jagiellonian University Medical College, Medyczna 9, 30-688 Cracow, Poland; michal.abram@doctoral.uj.edu.pl (M.A.); marcin.jakubiec@doctoral.uj.edu.pl (M.J.); rafa1.kaminski@uj.edu.pl (R.M.K.); 2 Department of Pharmacodynamics, Faculty of Pharmacy, Jagiellonian University Medical College, Medyczna 9, 30-688 Cracow, Poland; a.rapacz@uj.edu.pl (A.R.); szczepan.mogilski@uj.edu.pl (S.M.); 3 Department of Technology and Biotechnology of Drugs, Faculty of Pharmacy, Jagiellonian University Medical College, Medyczna 9, 30-688 Cracow, Poland; gniewomir.latacz@uj.edu.pl

**Keywords:** hybrid molecules, pyrrolidine-2,5-dione, anticonvulsant activity, antinociceptive activity, in vitro binding/functional studies, absorption, distribution, metabolism, excretion, toxicity (ADME-Tox) properties

## Abstract

Epilepsy belongs to the most common and debilitating neurological disorders with multifactorial pathophysiology and a high level of drug resistance. Therefore, with the aim of searching for new, more effective, and/or safer therapeutics, we discovered a focused series of original hybrid pyrrolidine-2,5-dione derivatives with potent anticonvulsant properties. We applied an optimized coupling reaction yielding several hybrid compounds that showed broad-spectrum activity in widely accepted animal seizure models, namely, the maximal electroshock (MES) test and the psychomotor 6 Hz (32 mA) seizure model in mice. The most potent anticonvulsant activity and favorable safety profile was demonstrated for compound **30** (median effective dose (ED_50_) MES = 45.6 mg/kg, ED_50_ 6 Hz (32 mA) = 39.5 mg/kg, median toxic dose (TD_50_) (rotarod test) = 162.4 mg/kg). Anticonvulsant drugs often show activity in pain models, and compound **30** was also proven effective in the formalin test of tonic pain, the capsaicin-induced pain model, and the oxaliplatin (OXPT)-induced neuropathic pain model in mice. Our studies showed that the most plausible mechanism of action of **30** involves inhibition of calcium currents mediated by Cav_1.2_ (L-type) channels. Importantly, **30** revealed high metabolic stability on human liver microsomes, negligible hepatotoxicity, and relatively weak inhibition of CYP3A4, CYP2D6, and CYP2C9 isoforms of cytochrome P450, compared to reference compounds. The promising in vivo activity profile and drug-like properties of compound **30** make it an interesting candidate for further preclinical development.

## 1. Introduction

Epilepsy remains one of the least understood neurological diseases with complex and multifactorial pathogenesis. It is estimated that about 50 million people worldwide suffer from this disease, making it the second most common neurological disorder after stroke [1,2]. Despite unquestionable advances in epilepsy research, nearly 30% of patients still experience uncontrolled, debilitating seizures [3]. Furthermore, even if the desired therapeutic effect is achieved, most of the available antiepileptic drugs (AEDs) often produce problematic side effects, e.g., memory impairment, diminished attention, and executive function or somnolence, due to their unspecific inhibitory influence on the central nervous system (CNS) [4]. In recent years, several new AEDs such as levetiracetam, brivaracetam, lacosamide, or perampanel have been introduced into the pharmacotherapy of seizures. However, despite their efficacy in several types of epilepsy, as well as better tolerance by patients, compared to older AEDs, they have not resolved the problem of drug-resistant epilepsy [5].

The multitargeted compounds which usually show broad pharmacological activity seem to be especially relevant for diseases with multifactorial origin and conditions with a high risk of drug resistance, such as depression, cancer, Parkinson’s disease, Alzheimer’s disease, neuropathic pain, and epilepsy [6,7,8,9]. Substances characterized by multitarget activity typically include hybrid or chimeric structures that contain several pharmacophores merged as one chemical scaffold [10]. This “multistructural” chemical composition gives the possibility of interaction with different biological targets potentially translating into better therapeutic efficacy. The most essential advantages of multitargeted compounds vs. combination therapies, which are usually required in severe diseases, include reduced risk of drug–drug interactions, limited side effects, and better patient compliance [11].

Our previous studies proved that the application of a molecular hybridization strategy may result in anticonvulsants with a wide spectrum of activity in different animal models of epilepsy [9,12,13,14,15]. Impotently, a number of such molecules also displayed potent antinociceptive activity in the in vivo studies. These compounds were designed as hybrid molecules on the basis of the pyrrolidine-2,5-dione core ring that merges structural fragments of known and clinically used AEDs, namely, ethosuximide (ETX), levetiracetam (LEV), and lacosamide (LCS). The in vivo data revealed that the several hybrid substances showed distinctly more potent and/or wider anticonvulsant efficacy compared to each of the aforementioned AEDs. In consequence, the hybrid compounds reported previously [9,12,13,14,15] were active in all three seizure models, i.e., the maximal electroshock (MES), subcutaneous pentylenetetrazole (*sc*PTZ), and the psychomotor 6 Hz (32 mA) seizure model. In contrast, ETX protected mice only in the *sc*PTZ model, while LCS was active in the MES and 6 Hz seizure models; lastly, LEV showed efficacy only in the 6 Hz seizure model. Thus, it is postulated that the proposed multifunctional compounds may be potentially effective in a broad range of human epilepsies characterized by tonic–clonic seizures, absence seizures, and importantly the most common focal seizures that affect more than 50% patients [16].

**Figure 1:**
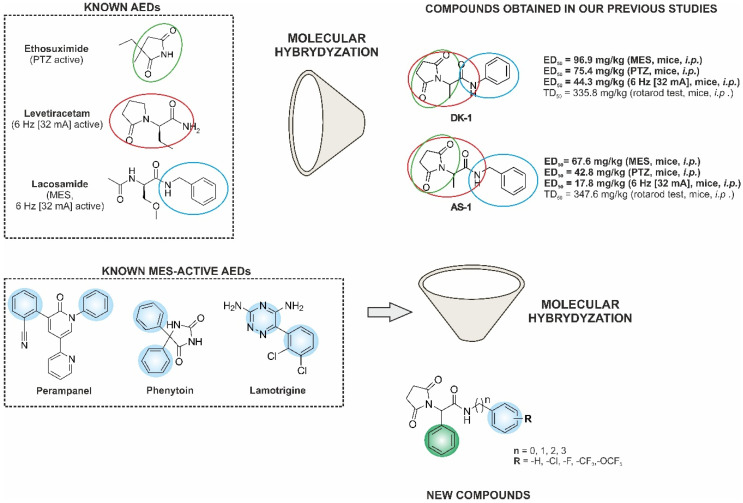
Design and general structure of hybrid 2-(2,5-dioxopyrrolidin-1-yl)-2-phenylacetamide derivatives (the main modification site is marked in green).

The MES test is still recognized as one of the most useful experimental tools for early identification of new AEDs, and it is recognized as an animal model of tonic–clonic seizures in human epilepsy [17]. The structure–activity relationship analysis of known AEDs that show potent protection in the MES test revealed that the majority of these compounds contain two aromatic rings in their structure (e.g., lamotrigine, phenytoin, or perampanel; see Figure 1). Therefore, we aimed to obtain new hybrid compounds with increased protection in the MES test; we report herein a series of new 2-(2,5-dioxopyrrolidin-1-yl)-2-phenylacetamides. These molecules were designed as analogues of previously described 2-(2,5-dioxopyrrolidin-1-yl)-2-alkylacetamides with an additional benzene ring attached to the side methyl group (marked in green, Figure 1). It should be stressed here that the aforementioned 2-(2,5-dioxopyrrolidin-1-yl)-2-alkylacetamides displayed only weak activity in the MES test [12,13]. Furthermore, we also hypothesized that the introduction of an additional aromatic moiety resulting in an increased lipophilicity may afford better blood–brain barrier penetration and more potent activity. As the amine function, we introduced unsubstituted phenylamine, benzylamine, phenylethylamine, and phenylpropylamine or their selected derivatives containing exclusively electron-withdrawing atoms or groups that appeared to be essential for anticonvulsant activity on the basis of previous results [12,13]. The design process and the general structure of the compounds described in the current paper are shown in Figure 1. For the selected most potent anticonvulsant compounds, we also assessed their antinociceptive properties in vivo, as well as performed mechanism-of-action studies and characterized basic absorption, distribution, metabolism, excretion, toxicity (ADME-Tox) properties in vitro.

## 2. Results and Discussion

### 2.1. Chemistry

**Scheme 1:**
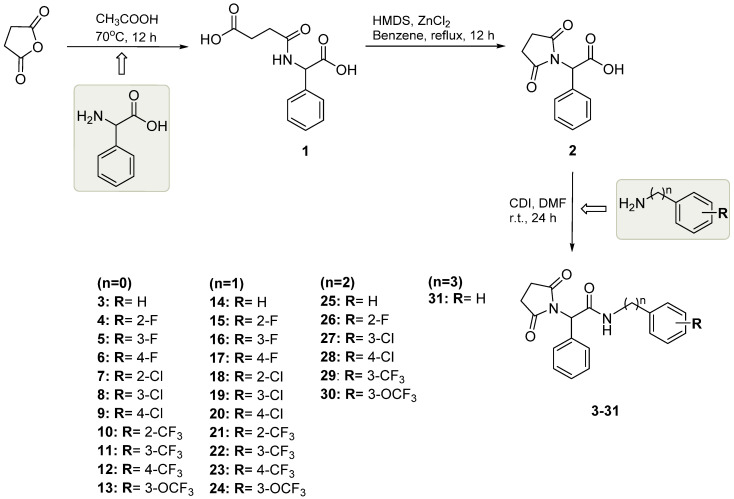
Synthesis of intermediates **1**, **2** and target compounds **3**–**31**.

Compounds **3**–**31** were synthesized using a multistep procedure according to Scheme 1. First, succinamic acid (**1**) was obtained by reacting equimolar amounts of commercially available succinic anhydride with dl-phenylglycine, and this reaction was carried out in 70 °C in acetic acid. Succinamic acid (**1**) was obtained as a solid substance after wash up with diethyl ether and was used without future purification. Next, the hexamethyldisilazane (HMDS)-promoted cyclization reaction of **1**, performed in benzene according to previously reported method [18], yielded monocarboxylic acid (**2**). The final compounds **3**–**31** were obtained by coupling of intermediate **2** with respective primary amine derivatives in the presence of carbonyldiimidazole (CDI) as the coupling reagent in dry dimethylformamide (DMF) as a solvent. This reaction was carried out at room temperature, and its progress was monitored by high-performance liquid chromatography (HPLC) (completion at ~24 h). Final compounds were purified by flash column chromatography and were obtained as solid substances, followed by concentration of organic solvents under reduced pressure. The target hybrid compounds were obtained with good yield (62.9–79.2%). The structures of intermediates and final molecules were confirmed by ^1^H-NMR, ^13^C-NMR (selected molecules), and/or LC–MS spectral analysis. Elemental analyses (C, H, and N) were performed for all the final compounds synthesized. The detailed physicochemical and spectral data are summarized in Section 3.

In the case of compounds **24** and **30**, the respective noncommercial primary amines **A1** and **A2** were synthesized prior to the coupling reaction. Amines **A1** and **A2** were obtained via reduction of commercially available nitriles using the solid LiAlH_4_ as a reductive agent (Scheme S1, Supplementary Materials). At first, the tetrahydrofuran (THF) solution of the given nitrile derivative was added dropwise to the suspension of LiAlH_4_ in anhydrous THF at 0 °C. Afterward, the reaction mixture was stirred at room temperature for additional 6 h. The progress of the reaction was monitored by HPLC. After the reaction was completed, the excess of LiAlH_4_ was neutralized by addition of 10% NaOH at 0 °C. Next, the reaction mixture was filtered through Celite 545 (Merck, Darmstadt, Germany), and the obtained solution was extracted with dichloromethane (DCM). The organic layer was dried over anhydrous Na_2_SO_4_, and then evaporated to dryness. Amines **A1** and **A2** were used to further reactions without purification. The detailed synthetic procedure and the physicochemical and spectral data for **A1** and **A2** are described in the Supplementary Materials.

### 2.2. Anticonvulsant Activity

Empirical screening in animal models of human epileptic seizures is still recognized as a standard and effective paradigm in the discovery and development of new anticonvulsants [19]. Notably, this approach enabled identification of nearly all commercially launched AEDs so far. It should also be stressed that the screening approach enables detection of both substances acting on molecular targets that are well known for existing anticonvulsants, as well as compounds with new and undefined modes of action. In consequence, all final compounds **3**–**31** were initially evaluated in the MES test, which is an experimental model of tonic–clonic epilepsy [17]. This test, as a mechanism-independent animal seizure model, detects compounds capable of preventing the spread of seizures when all neurons in the brain are maximally active. It is noteworthy that the MES test is still recognized as the most useful preclinical seizure model and, hence, is widely employed during the early identification of new AED candidates. Importantly, the MES test may also help to find substances with unique mechanism of action [20].

Bearing in mind the aforementioned facts, all final substances were initially studied in the MES test after intraperitoneal (i.p.) administration at a fixed dose of 100 mg/kg in mice (in a group consisting of four animals). The protection effect against MES seizures was observed at 0.5 h post intraperitoneal (i.p.) injection. The pretreatment time was chosen on the basis of our previous experimentations [9,12,13,14,15]. According to the screening data (Tables S1–S3, Supplementary Materials), at least 50% protection, which was considered as satisfactory, was demonstrated for compounds **3**, **4**, **6**, **8**, **9** (anilinamide derivatives), **14**, **19**, **21**, **22**, **24** (benzylamide derivatives), **25**, **27**–**30** (phenyletylamide derivatives), and **31** (phenylpropanamide analogue). Notably, the maximal (100%) protection was observed for 2-F (**4**), 3-CF_3_ (**29**), and 3-OCF_3_ (**30**) congeners. During the next step of pharmacological characterization, all MES-active substances were tested in the mouse 6 Hz (32 mA), an animal model of human focal seizures [21] (for details, see Tables S1–S3, Supplementary Materials). The data obtained revealed that 12 molecules (**3**, **6**, **14**, **19**, **22**, **24**, **25**, **27**–**31**) protected at least 50% of mice in this seizure test. The full (100%) activity was observed only for 3-OCF_3_ benzylamide derivative **24**, whereas 75% efficacy was shown for **3**, **25**, and **27–30**. According to the screening results, 11 compounds may be considered as broad-spectrum anticonvulsants that are effective in both MES and 6 Hz (32 mA) seizure models, i.e., **3**, **6**, **14**, **19**, **22**, **24**, **25**, **27**–**31**. In general, phenylethylamide derivatives **25** (H), **28** (4-Cl), **29** (3-CF_3_), and **30** (3-OCF_3_), and benzylamide derivative **24** (3-OCF_3_) displayed the most beneficial anticonvulsant properties (i.e., the highest percentage of protection in each seizure model). A narrower spectrum of protection was observed for anilinamides, which were effective predominantly in the MES test (excluding **3** and **6**). In parallel to the anticonvulsant screening, all the aforementioned active compounds were studied in the rotarod test to assess their influence on motor coordination of mice. In this assay, none of substances produced acute neurological toxicity at dose of 100 mg/kg (in some cases, only minimal effects were seen). These results suggest a distinct separation between anticonvulsant effective and toxic doses (i.e., inducing motor deficits), which was confirmed in the in vivo quantitative investigations (for details, see Table 1). According to the aforementioned initial screening data, it may be concluded that the most potent and broad anticonvulsant activity seemed to be provided by the phenylethylamide derivatives. Taking into consideration the optimal substitution mode of the aromatic ring, it can also be concluded that (1) the unsubstituted phenyl rings are preferential for activity in each series, (2) the small substituents such as chlorine or fluorine atoms are preferential in the case of anilinamide derivatives, and (3) chlorine atoms and large trifluoromethyl and trifluoromethoxy groups seem to be more beneficial for activity among benzylamides and phenylethylamides.

In the next step of in vivo characterization, we determined the median effective doses (ED_50_) for all anticonvulsants protecting 50% or more mice in each seizure model (MES or/and 6 Hz (32 mA)), as well as the median toxic doses (TD_50_) in the rotarod test at the 0.5 h time point. Both the aforementioned parameters enabled calculation of the protective indexes (PIs), which are a measure of potential therapeutic window of the drug candidate. In parallel, we performed a series of comparative studies using reference compounds, e.g., lacosamide (LCS), lamotrigine (LTG), and valproic acid (VPA), which are active in the MES and 6 Hz (32 mA) seizure tests. The obtained results are summarized in Table 1.

As expected on the basis of screening data, phenylethylamides **27**, **29**, and **30** displayed the most potent activity in the MES and/or 6 Hz (32 mA) seizure tests. Satisfactory protection in the MES test (50%) was shown for a benzylamide derivative **21** (this compound was unfortunately not effective in the 6 Hz test). In general, more potent activity was observed in the 6 Hz (32 mA) seizure model, and the lowest ED_50_ values were obtained with **6**, **14**, **22**, **24**, **27**, **29**, and **30**. It should be emphasized that all anilinamides (**3**, **4**, **6**, **8**, **9**) and benzylamides (**14**, **19**, **21**, **22**, **24**) did not impair motor coordination of mice at doses as high as 300 mg/kg. Phenylethylamides **25**, **28**–**30** displayed moderate acute neurological toxicity (at doses around or below 200 mg/kg). Among the whole series of hybrid derivatives, the most potent protection in both seizure models and moderate but acceptable acute neurological toxicity were obtained with **30**, which was identified as a lead compound. The comparison of data obtained with the test compounds and reference AEDs (VPA in particular) indicated a more potent protection, as well as a predominantly more beneficial safety margin (expressed as PIs), for the majority of the hybrid derivatives. It should be emphasized that VPA is still recognized as the most frequently prescribed and the most effective first-line AED in different types of epilepsies [22]. However, potent activity in both seizure models and beneficial PI values were observed for LCS and LTG, which act as sodium channel blockers.

In summary, the proposed structural modification relying on the introduction of the additional phenyl moiety in the acetamide linker did not increase activity in the MES test compared to aliphatic analogues represented by maternal compounds DK-1 and AS-1 (see Figure 1), which was the main hypothesis behind the current series of compounds. It should be stressed, however, that several substances (e.g., **21**, **24**, **27**, **29**, and **30**) revealed beneficial anticonvulsant activity, with an acceptable safety profile; thus, they may be subjects for further structural optimization.

### 2.3. Antinociceptive Activity

AEDs are also commonly used in the treatment of neuropathic pain and other central pain conditions. Therefore, with an aim of further pharmacological characterization of compounds described herein, we decided to study their antinociceptive properties in the in vivo assays. These studies were performed for two representative molecules with different anticonvulsant profiles, namely, compound **4**, which showed protection exclusively in the MES test, and **30**, which was active in both MES and 6 Hz (32 mA) seizure models. At first, both aforementioned compounds were tested in the formalin-induced tonic pain model, which is recognized as a valuable screening tool [24]. In this test, the injection of formalin into the plantar surface of the mouse paw produces specific behaviors like paw lifting, flinching, and licking. Furthermore, the nociceptive response is biphasic where, in phase I (0–5 min), pain results from the direct activation of primary nociceptive afferents, and, in phase II (10–30 min), pain results mainly from the central sensitization in the dorsal horn of the spinal cord [25]. Moreover, it has been reported that formalin induces pathological changes that resemble those observed in nerve injury and neuropathic pain [24]. Thus, this test has been successfully used to assess the analgesic efficacy of a variety of compounds such as opioids, nonsteroidal anti-inflammatory drugs (NSAIDs), corticosteroids, and *N*-methyl-d-aspartate (NMDA) receptor antagonists, as well as AEDs such as carbamazepine, topiramate, or gabapentin [26,27].

**Figure 2:**
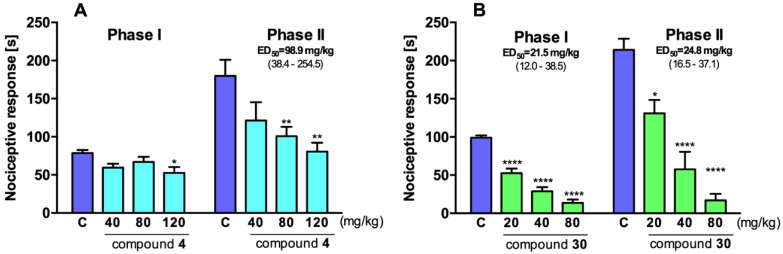
Antinociceptive activity of compounds **4** (**A**) and **30** (**B**) in the formalin test. Results are shown as time of licking in phase I (0–5 min after intraplantar injection of formalin) and in phase II (15–30 min after formalin injection). Each value represents the mean ± standard error of the mean (SEM) for 8–10 animals. Statistical analysis: one-way ANOVA followed by post hoc Dunnett’s test. Statistical significance compared to vehicle-treated animals (Tween): * *p* < 0.05, ** *p* < 0.01, **** *p* < 0.0001. C—control group.

As mentioned above, we investigated the analgesic properties of two of the most promising and interesting compounds: **4** and **30**. As shown in Figure 2, the i.p. administration of compound **4** prior to the subcutaneous injection of formalin attenuated the nociceptive response in mice in both phases of the test; however, in the first phase, the activity was statistically significant only at the highest dose of 120 mg/kg. It was slightly more efficient in the second phase of the model with an ED_50_ value equal to 98.9 mg/kg. In contrast, compound **30** was much more effective (almost four times more potent) in both phases (phase I: ED_50_ = 21.5 mg/kg and phase II: ED_50_ = 24.8 mg/kg). Particularly interesting is the activity of the compound in the first phase, since it suggests its efficacy with regard to the pain associated with acute and direct damage/disfunction of the nerve endings. These results showed favorable analgesic properties, especially for compound **30**, which were subsequently confirmed in other models of pain such as capsaicin-induced neurogenic pain and oxaliplatin (OXPT)-induced neuropathic pain in mice.

**Figure 3:**
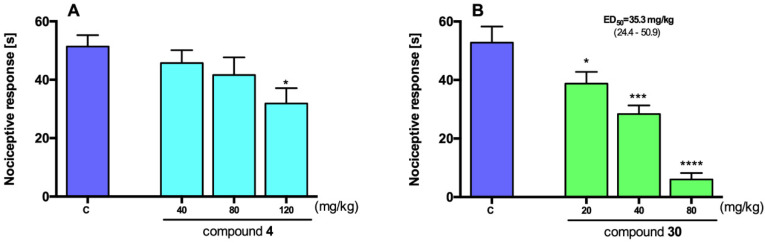
Antinociceptive activity of compounds **4** (**A**) and **30** (**B**) in the capsaicin test. Results are shown as time of licking in the period of 5 min after intraplantar injection of capsaicin. Each value represents the mean ± SEM for 8–10 animals. Statistical analysis: one-way ANOVA followed by post hoc Dunnett’s test. Statistical significance compared to vehicle-treated animals (Tween): * *p* < 0.05, *** *p* < 0.00, **** *p* < 0.0001. C—control group.

We used the capsaicin-induced model of pain to test the potential of the compounds to inhibit the neurogenic (acute) response to noxious stimuli, which was suggested by their activity in the first phase of the formalin test. Capsaicin activates C polymodal fibers and enhances their firing, which contributes to subsequent hyperalgesia. The molecular mechanism of action of capsaicin is the activation of the nonselective cation channel, transient receptor potential vanilloid-1 (TRPV1), which is localized on nociceptive free nerve endings. That induces the release of proinflammatory neuropeptides such as calcitonin gene-related peptide (CGRP), vasoactive intestinal peptide (VIP), and substance P (SP). The phenomenon of the induction of tissue inflammation by the peripheral nervous system is called neurogenic inflammation, and it is involved in the development of inflammatory and neuropathic pain. AEDs such as gabapentin, topiramate, or lamotrigine decrease capsaicin-induced nocifensive behavior [25]. We observed a similar effect especially in the case of compound **30**, which significantly attenuated the nociceptive response induced by capsaicin in a dose-dependent manner (Figure 3B). Its ED_50_ value was found to be 35.3 mg/kg. Compound 4 was less active and was able to significantly attenuate nociceptive response in capsaicin-induced pain by 37.9%, but only at the highest applied dose of 120 mg/kg (Figure 3A). The obtained results show that the test compound 30 has the potential for inhibiting acute, neurogenic pain, probably by stabilizing the firing polymodal fibers and/or central mechanisms of analgesia.

**Figure 4:**
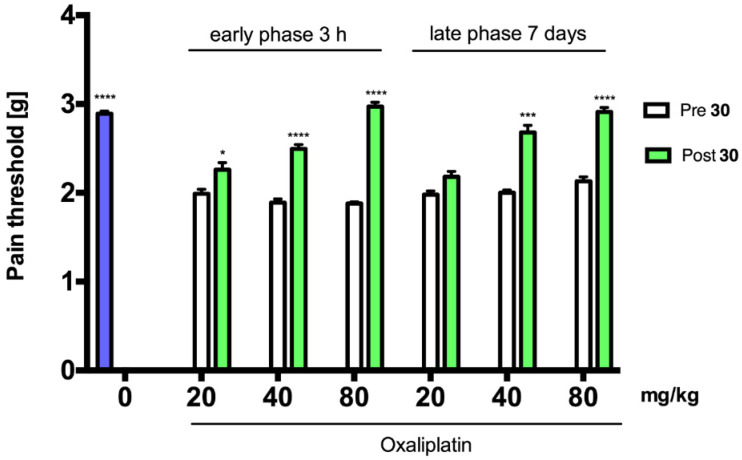
Antiallodynic effects of compound **30** administered at the doses of 20 mg/kg, 40 mg/kg, and 80 mg/kg in the tactile allodynia evaluated in the von Frey test in the oxaliplatin (OXPT)-induced model of peripheral neuropathy. Results compared to vehicle-treated group (pre **30**). Statistical analysis: repeated measures analysis of variance (ANOVA), followed by Dunnett’s post hoc comparison: * *p* < 0.05, *** *p* < 0.001, **** *p* < 0.0001.

In order to investigate the analgesic activity of the most promising compound **30** in a neuropathic pain model, we tested it in oxaliplatin (OXPT)-induced peripheral neuropathy, in which mechanical and cold allodynia result from impaired function of ion channels, among others [28]. The early phase of neuropathic pain appears hours after OXPT administration, while the late phase develops days after OXPT. Thus, we tested compound **30** 3 h and 7 days after the injection of OXPT. We investigated the influence of compound **30** on tactile allodynia using the von Frey method. Three hours after the administration of OXPT we observed a significant decrease in the mean value of force that caused paw withdrawal reaction (1.89 ± 0.04 g) (64.2% of the baseline) compared to nontreated animals (2.94 ± 0.06 g) (the baseline). Seven days after OXPT administration, the pain threshold was 2.00 ± 0.03 (68.0% of the baseline). The observed tactile allodynia was attenuated by compound **30**, which elevated pain sensitivity threshold in the early phase in a dose-dependent manner (Figure 4) (2.26 ± 0.08 g, 2.49 ± 0.05 g, and 2.97 ± 0.05 g at the dose of 20 mg/kg, 40 mg/kg, and 80 mg/kg, respectively, which correspond to 76.8%, 84.7%, and 101.0% of the baseline value). In the late phase, the values of pain threshold were as follows 2.18 ± 0.06 g, 2.60 ± 0.08 g, and 2.91 ± 0.05 g, which correspond to 74.1%, 88.4%, and 98.9% of the baseline value. These results indicate that compound **30** has the potential for relieving neuropathic pain.

### 2.4. In Vitro Radioligand Binding Studies and Functional Assays

The previous studies in series of 2-(2,5-dioxopyrrolidin-1-yl)propanamides, represented by compounds AS-1 and DK-1 (see Figure 1), showed that these molecules interact with voltage-gated sodium channels and Cav_1.2_ (L-type) calcium channels at very high concentrations of 100 µM or 500 µM [12]. Taking into consideration the structural similarities of molecules described in the current manuscript with their chemical prototypes AS-1 and DK-1, the binding studies toward aforementioned molecular targets were performed commercially for several active substances from each subseries (**3**, **4**, **21**, **24**, **25**, **27**, **29**, and **30**). Furthermore, we also tested two other and well-known targets for AEDs, namely, the Cav_2.2_ (*N*-type) calcium channel and *gamma*-aminobutyric acid (GABA)-A receptor. With the aim of excluding potentially dangerous proarrhythmic properties for each compound, their interaction with the potassium (hERG) channel was also tested. Notably, the potassium (hERG) channel is recognized as one of the most important off-targets, and the potential effects on this ion channel should be eliminated in early stages of the new drug development. The details are shown in Table 2.

The results of the binding studies showed that only four compounds **24**, **27**, **29**, and **30** revealed moderate binding to voltage-gated calcium channels Cav_1.2_ at a concentration of 10 µM. Importantly, these substances were also identified as the most effective anticonvulsants with potent protection especially in the 6 Hz (32 mA) seizure model. Thus, the interaction with Cav_1.2_ channels seems to be at least partially responsible for activity in the aforementioned model of focal seizures. These observations are consistent with results obtained in our previous studies that clearly indicate on Cav_1.2_ channel antagonism as an important mechanism underlying anticonvulsant properties [13,14,15]. Notably, it is well known that Cav_1.2_ channels are widely localized in the CNS neurons, predominantly on post-synaptic parts; consequently, they regulate neuronal firing [29]. It should be emphasized that the most current neurobiological studies implicate Cav_1.2_ channels in the pathogenesis of chronic and neuropathic pain [30]. Thus, antagonists of these ion channels may be considered as promising candidates for new antinociceptive drugs [31]. The functional assays applying the cell-based flux studies performed for compounds **24**, **27**, **29**, and **30** showed their moderate antagonist properties (Table 3).

Interestingly, none of the tested compounds interacted with voltage-gated sodium channels, Cav_2.2_ calcium channels, GABA-A receptors, and especially the potassium (hERG) channel at concentrations as high as 100 µM. The latter result is particularly important since it minimizes the risk of potential proarrhythmic activity of these compounds. Summing up, on the basis of the binding and functional studies, it may be concluded that Cav_1.2_ channel antagonism is probably involved in the mechanism of action for the compounds reported herein. Nevertheless, it should be stressed that the identification of additional and currently undefined molecular targets responsible for the biological properties is highly possible during further in vitro characterization.

### 2.5. In Vitro ADME-Tox Assays

Long-term use of AEDs may often lead to numerous adverse effects both acute (affecting the CNS and the gastrointestinal tract) and chronic (including neuropsychiatric disorders or gastrointestinal tract, endocrine, hematologic, liver, metabolic, dermatologic, and systemic symptoms) [32], as well as drug–drug interactions (DDIs), especially with the metabolic origin mediated by different isoforms of cytochrome P450 (CYP). Until now, 10 CYP isoforms have been identified in a typical human liver, and six of them (CYP1A2, CYP2C8, CYP2C9, CYP2C19, CYP2D6, and CYP3A4) appear to be engaged in biotransformation of most drugs. Notably, among these isoforms, CYP3A4, CYP2D6, and CYP2C9 are responsible for the metabolism of more than 50% of marketed drugs and the associated DDIs. The induction or inhibition of these P450 isotypes may influence the metabolic clearance and blood concentration of co-administered drugs, resulting in lower therapeutic efficacy or toxic drug effects, respectively. The problems related with the DDIs are especially visible for the first generation of AEDs which may interact with different CYP isoforms. The clearest examples of these adverse effects are phenytoin, phenobarbital, and carbamazepine which are known as CYP3A4, CYP2C9, CYP1A2, and CYP2B6 inductors, and valproic acid, recognized as a CYP2C9 inhibitor. Notably, the DDIs are described also for the newest AEDs, e.g., cannabidiol (CBD) and clobazam. Clobazam, during the biotransformation, is *N*-demethylated by CYP3A4 and, to a lesser extent, by CYP2C19 and CYP2B. The main metabolite, *N*-desmethylclobazam (or norclobazam), is hydroxylated next by CYP2C19. The DDI between CBD and clobazam leading to a significant increase in the level of *N*-desmethylclobazam results from inhibition of CYP2C19 activity by CBD [33]. It should also be stressed herein that some AEDs (including the newest preparations) form toxic metabolites during biotransformation, e.g., the reactive metabolite of felbamate causing aplastic anemia [34] or the 10,11-epoxide of carbamazepine, which facilitates its and the parent drug’s metabolism (autoinduction) [35]. Consequently, the evaluation of CYPs induction/inhibition or hepatotoxic potential of new biologically active compounds is crucial at an earlier stage of drug development.

Considering the aforementioned facts, drug-like properties of the most promising compounds from each subseries (**4**, **24**, **30**) were estimated next within this study. Standard in vitro methods were used for ADME-Tox parameter evaluation. All tests were performed acording to descrived previously protocols [14,15,36].

#### 2.5.1. Parallel Artificial Membrane Permeability Assay (PAMPA) 

Passive diffusion was tested in the parallel artificial membrane permeability assay (PAMPA) and expressed as the permeability coefficient (*Pe*). A highly permeable compound, caffeine (CFN), and a poorly permeable compound, norfloxacin (NFX), were used as the references. All compounds selected for this assay (**4, 24** and **30**) showed very high ability to passively diffuse through the biological membranes with *Pe* values close to or even higher (compound **30**) than CFN (Table 4).

#### 2.5.2. Metabolic Stability

Human liver microsomes (HLMs) were used for metabolic stability determination. Moreover, the most probable phase I reactions and structures of metabolites were proposed according to ion fragment analyses with the in silico support of MetaSite 6.0.1 software. In general, pyrrolidine-2,5-dione derivatives displayed satisfied stability as their conversion into metabolites was lower than 30% during 120 min incubation with HLMs. These results correlate with our previous studies, where high metabolic stability within this chemical group was also found [9,12,14,15,37]. Compound **4** showed the highest susceptibility for enzymatic biotransformations (71.4% of parent compound remained in the reaction mixture; see Figure S1, Supplementary Materials), resulting in double-hydroxylations (**M1**), pyrrolidine-2,5-dione ring hydrolysis (**M2**), or monohydroxylation (**M3**) as the most probable metabolic pathways (Figures S2 and S7, Supplementary Materials). On the other hand, an excellent metabolic stability of compound **24** was observed. Less than 4% of this derivative was converted into double-hydroxylated metabolite **M1** (Figures S3, S4, and S7, Supplementary Materials). Finally, two monohydroxylated metabolites (**M1**, **M2**) were found in the reaction mixture with HLMs and **30**, and around 17% of this compound was metabolized (Figures S5–S7, Supplementary Materials).

#### 2.5.3. Influence on Function of Cytochrome P450 Isoforms—CYP3A4, CYP2D6, and CYP2C9

**Figure 5:**
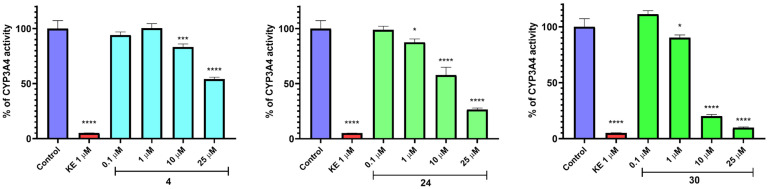
The influence of **4**, **24**, **30**, and reference inhibitor ketoconazole (KE) on CYP3A4 activity. Statistical significance (* *p* < 0.05, *** *p* < 0.001, **** *p* < 0.0001) was analyzed by Graph Pad Prism 8.0.1 software using one-way ANOVA and Bonferroni’s multiple comparison post test. The compounds were examined in triplicate.

The luminescent CYP3A4, 2D6, and 2C9 P450-Glo assays were used for the prediction of DDIs risk. In case of CYP3A4, all tested derivatives statistically significantly inhibited its activity at 10 µM. The highest inhibition was observed for derivatives with trifluoromethoxy substituent **24** and **30**. These compounds still slightly inhibited CYP3A4 activity (*p* < 0.05) even at low concentration (1 µM). However, this effect was much weaker than that observed for 1 µM of the reference CYP3A4 inhibitor ketoconazole (KE) (Figure 5)

**Figure 6:**
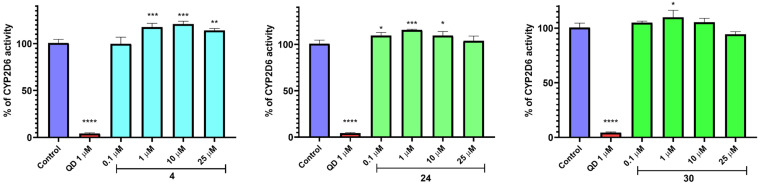
The influence of **4**, **24**, **30**, and reference inhibitor quinidin (QD) on CYP2D6 activity. Statistical significance (* *p* < 0.05,** *p* < 0.01, *** *p* < 0.001, **** *p* < 0.0001) was analyzed by Graph Pad Prism 8.0.1 software using one-way ANOVA and Bonferroni’s multiple comparison post test. The compounds were examined in triplicate.

Furthermore, slight but statistically significant activation of CYP2D6 was observed for all compounds, whereas quinidine (QD) used here as the reference inhibited this isoform entirely at 1 µM (Figure 6).

**Figure 7:**
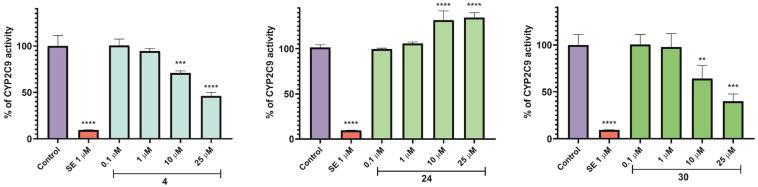
The influence of **4**, **24**, **30** (10 µM) and the reference inhibitor sulfaphenazole (SE) on CYP2C9 activity. Statistical significance (** *p* < 0.01, *** *p* < 0.001, **** *p* < 0.0001) was analyzed by Graph Pad Prism 8.0.1 software using one-way ANOVA and Bonferroni’s multiple comparison post test. The compounds were examined in triplicate.

Surprisingly, the opposite effect on CYP2C9 was observed within this group, whereby compounds **4** and **30** blocked its activity at the highest concentrations of 10 and 25 µM, whereas the same doses of **24** activated this CYP isoform up to 140% of control. However, the observed inhibition effect of **4** and **30** was found to be weaker in comparison to the reference inhibitor sulfaphenazole (SE), which almost completely blocked CYP2C9 activity at 1 µM (Figure 7).

#### 2.5.4. Determination of Hepatotoxicity Risk

**Figure 8:**
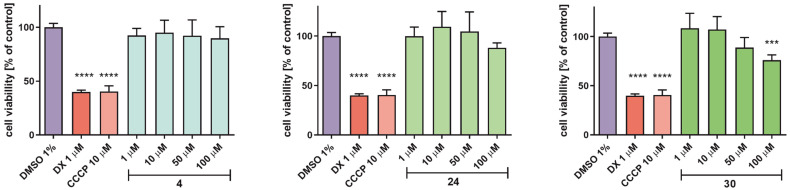
The effect of **4**, **24**, **30**, and cytostatic drug doxorubicin (DX) and the mitochondrial toxin carbonyl cyanide 3-chlorophenyl-hydrazone (CCCP) on hepatoma HepG2 cell lines viability after 72 h of incubation at 37 °C, 5% CO_2_. The statistical significance (GraphPad Prism 8.0.1) was evaluated by a one-way ANOVA, followed by Bonferroni’s comparison test (*** *p* < 0.001, **** *p* < 0.0001) compared with negative control dimethyl sulfoxide (DMSO) 1% in growth medium).

The hepatotoxicity assessment was done with use of hepatoma HepG2 cells, which were incubated for 72 h in the presence of 1–100 µM concentrations of **4**, **24**, and **30**. The CellTiter 96^®^ aqueous nonradioactive cell proliferation assay (MTS) purchased from Promega (Madison, WI, USA) was used for determination of cell viability. The absorbance was measured using a microplate reader EnSpire (PerkinElmer, Waltham, MA, USA) at 492 nm. The obtained results confirmed the safety of the investigated series. The statistically significant (*p* < 0.001) effect on cells viability was determined for compound **30**, but only at the very high concentration of 100 µM (Figure 8).

Overall, the results obtained in the in vitro ADME-Tox assays indicate satisfactory drug-like properties of all tested compounds (**4**, **24**, and **30**). In particular, very high (similar to CFN) passive diffusion in PAMPA, excellent metabolic stability, and low hepatotoxic effects were confimed. In general, DDI assays showed potential interactions of tested derivatives with all CYP isoforms used. However, the observed effects were much weaker than when using the reference inhibitors KE, QD, and SE.

## 3. Materials and Methods

### 3.1. Chemistry

All chemicals and solvents were purchased from Sigma-Aldrich (St. Louis, MO, USA) and were used without further purification. Melting points (m.p.) were determined in open capillaries on a Büchi 353 melting point apparatus (Büchi Labortechnik, Flawil, Switzerland). Thin-layer chromatography (TLC) and gradient UPLC chromatography were used to assess the purity and homogeneity of the compounds. TLC was carried out on silica gel 60 F_254_ pre-coated aluminum sheets (Macherey-Nagel, Düren, Germany), using the following developing systems: S_1_–DCM:MeOH (9:0.3; *v*/*v*), S_2_–DCM:MeOH (9:0.5; *v*/*v*), S_3_–DCM:MeOH (9:0.7; *v*/*v*), S_4_–DCM:MeOH (9:1; *v*/*v*). Spot detection was carried out using ultraviolet (UV) light (λ = 254 nm). The UPLC and mass spectra (LC–MS) were obtained on a Waters ACQUITY™ TQD system (Waters, Milford, CT, USA) with the MS-TQ detector and UV–Vis–DAD eλ detector. The ACQUITY UPLC BEH C18, 1.7 µm (2.1 × 100 mm) column was used with the VanGuard Acquity UPLC BEH C18, 1.7 µm (2.1 × 5 mm) (Waters, Milford, CT, USA). Standard solutions (1 mg/mL) of each compound were prepared in an analytical grade MeCN/water mixture (1:1; *v*/*v*). Conditions applied were as follows: eluent A (water/0.1% HCOOH), eluent B (MeCN/0.1% HCOOH), a flow rate of 0.3 mL/min, a gradient of 5–100% B over 10 min, and an injection volume of 10 µL. The UPLC retention times (*t*_R_) are given in minutes. The purity of target compounds determined by use of chromatographic UPLC method was ≥95%. Preparative column chromatography was performed using silica gel 60 (particle size 0.063–0.200; 70–230 Mesh ATM) purchased from Macherey-Nagel (Duren, Germany). Elemental analyses (C, H, and N) for final compounds were carried out by a micro method using the elemental Vario EI III Elemental analyzer (Hanau, Germany). The results of elemental analyses were within ±0.4% of the theoretical values. ^1^H-NMR and ^13^C-NMR spectra were obtained in CDCl_3_ or dimethyl sulfoxide (DMSO)-D_6_ in a Varian Mercury spectrometer (Varian Inc., Palo Alto, CA, USA), in DMSO-D_6_ operating at 300 MHz (^1^H-NMR) and 75 MHz (^13^C-NMR), or in a JEOL-500 spectrometer (JEOL USA, Inc. MA, USA), in CDCl_3_ operating at 500 MHz (^1^H-NMR) and 126 MHz (^13^C-NMR). Chemical shifts are reported in δ values (ppm) relative to tetramethylsilane (TMS) δ = 0 (^1^H), as an internal standard. The *J* values are expressed in Hertz (Hz). Signal multiplicities are represented by the following abbreviations: s (singlet), br. s (broad singlet), d (doublet), dd (doublet of doublet), dq (doublet of quartets), t (triplet), td (triplet of doublets), q (quartet), and m (multiplet).

#### 3.1.1. Synthetic Procedure for Succinamic Acid (**1**)

Succinic anhydride (5.0 g, 50 mmol, 1 equivalent (eq)) was dissolved in 50 mL of glacial acetic acid and, afterward, an equimolar amount of dl-phenylglycine (7.56 g) was added. The reaction mixture was heated at 70 °C and next stirred for 12 h. After this time, acetic acid was evaporated to dryness. The intermediate **1** was obtained as a solid substance after wash up with diethyl ether.

##### 4-((Carboxy(phenyl)methyl)amino)-4-oxobutanoic Acid (**1**)

White solid. Yield: 92% (11.54 g); m.p. 199.4–200.6 °C; TLC: R_f_ = 0.25 (S_4_); UPLC (purity 100%): *t*_R_ = 2.77 min. LC–MS (ESI): *m*/*z* calculated for C_12_H_13_NO_5_ (M + H)^+^ 252.08, found 252.1.

#### 3.1.2. Synthetic Procedure for Monocarboxylic Acid (**2**)

To a suspension of the appropriate succinamic acid **1** (45 mmol, 1 eq) in dry benzene (150 mL), ZnCl_2_ (6.13 g, 45 mmol, 1 eq) was added, and the mixture was heated to 80 °C. Next, a solution of HMDS (10.89 g, 14.14 mL, 67.5 mmol, 1.5 eq) in dry benzene (15 mL) was added dropwise over 30 min. The reaction mixture was refluxed for an additional 12 h and concentrated under reduced pressure. After concentration, the residual solid was dissolved in DCM and extracted with 0.1 M HCl (3 × 50 mL), water (3 × 50 mL), and saturated brine (3 × 50 mL). The organic layer was dried over anhydrous Na_2_SO_4_ and then evaporated to dryness. Intermediate **2** was obtained as a solid substance after wash up with diethyl ether.

##### 2-(2,5-Dioxopyrrolidin-1-yl)-2-phenylacetic Acid (**2**)

White solid. Yield: 91% (9.54 g); m.p. 198.3–199.7 °C; TLC: R_f_ = 0.46 (S_4_); UPLC (purity 100%): *t*_R_ = 3.41 min. LC–MS (ESI): *m*/*z* calculated for C_12_H_11_NO_4_ (M + H)^+^ 234.08, found 234.1. ^1^H-NMR (300 MHz, DMSO-D_6_) δ 2.73 (s, 4H, imide), 5.76 (s, 1H, CH-COOH), 7.26–7.35 (m, 3H, ArH) 7.36–7.45 (m, 2H, ArH), 13.22 (br. s, 1H, COOH).

#### 3.1.3. Synthetic Procedure for Target Compounds (**3**–**31**)

Carbonyldiimidazole (0.585 g, 3.6 mmol, 1.2 eq) was dissolved in 10 mL of anhydrous DMF. Afterward, the solution was added to intermediate **2** (3 mmol, 1 eq) dissolved in 10 mL of anhydrous DMF (while stirring). After 0.5 h, the appropriate amine (3 mmol, 1 eq) dissolved in 5 mL of anhydrous DMF was added dropwise. The mixture was stirred for approximately 24 h at room temperature and evaporated to dryness. Column chromatography was applied for purification of crude products, using the following developing systems: S_1_ (**3**–**20**), S_2_ (**21**–**31**). The desired compounds were obtained as solid substances followed by concentration of organic solvents under reduced pressure.

##### 2-(2,5-Dioxopyrrolidin-1-yl)-*N*,2-diphenylacetamide (**3**)

White solid, yield: 69.4%, (0.63 g); m.p. 155.8–156.3 °C; TLC: R_f_ = 0.52 (S_1_); UPLC (purity >99.9%): *t*_R_ = 5.46 min. LC–MS (ESI): *m*/*z* calculated for C_18_H_16_N_2_O_3_ (M + H)^+^ 309.12, found 309.2. ^1^H-NMR (300 MHz, CDCl_3_) δ 2.74 (s, 4H, imide), 5.90 (s, 1H, CH), 7.11 (d, *J* = 7.3 Hz, 1H, ArH), 7.24–7.30 (m, 2H, ArH), 7.36–7.46 (m, 5H, ArH), 7.52 (s, 1H, NH), 7.58–7.67 (m, 2H, ArH). ^13^C-NMR (75 MHz, CDCl_3_) δ 28.2, 59.2, 120.1, 124.9, 129.0, 129.5, 129.6, 129.9, 133.8, 137.0, 164.8, 176.6. Analysis calculated for C_18_H_16_N_2_O_3_ (308.34): C: 70.12, H: 5.23, N: 9.09; found C: 70.25, H: 5.37, N: 9.26.

##### 2-(2,5-Dioxopyrrolidin-1-yl)-*N*-(2-fluorophenyl)-2-phenylacetamide (**4**)

White solid, yield: 68.7%; (0.67 g) m.p. 106.6–107.1 °C; TLC: R_f_ = 0.51 (S_1_); UPLC (purity >99.9%): *t*_R_ = 5.52 min. LC–MS (ESI): *m*/*z* calculated for C_18_H_15_FN_2_O_3_ (M + H)^+^ 327.11, found 327.3. ^1^H-NMR (300 MHz, CDCl_3_) δ 2.78 (s, 4H, imide), 5.92 (s, 1H, CH), 6.98–7.11 (m, 3H, ArH), 7.42–7.47 (m, 3H, ArH), 7.62–7.68 (m, 3H, ArH, NH), 8.25 (td, *J* = 7.91, 1.76 Hz, 1H, ArH). ^13^C-NMR (75 MHz, CDCl_3_) δ 28.2, 59.3, 114.8 (d, *J* = 18.4 Hz), 121.7, 124.5, 125.0, 125.1, 125.6 (d, *J* = 10.4 Hz), 129.6, 129.7–129.9, 133.6, 152.5 (d, *J* = 243.0 Hz), 154.1–154.1, 164.7, 176.4. Analysis calculated for C_18_H_15_FN_2_O_3_ (326.33): C: 66.25, H: 4.63, N: 8.58; found C: 66.32, H: 4.71, N: 8.79.

##### 2-(2,5-Dioxopyrrolidin-1-yl)-*N*-(3-fluorophenyl)-2-phenylacetamide (**5**)

White solid, yield: 72.3%, (0.71 g); m.p. 148.2–149.1 °C; TLC: R_f_ = 0.55 (S_1_); UPLC (purity >99.9%): *t*_R_ = 5.81 min. LC–MS (ESI): *m*/*z* calculated for C_18_H_15_FN_2_O_3_ (M + H)^+^ 327.11, found 327.4. ^1^H-NMR (300 MHz, CDCl_3_) δ 2.75 (s, 4H, imide), 5.89 (s, 1H, CH), 6.75–6.83 (m, 1H, ArH), 6.96–7.00 (m, 1H, ArH), 7.20 (td, *J* = 8.2, 6.5 Hz, 1H, ArH), 7.35–7.45 (m, 4H, ArH), 7.57–7.62 (m, 3H, ArH, NH). ^13^C-NMR (75 MHz, CDCl_3_) δ 28.2, 59.2, 107.6 (d, *J* = 26.5 Hz) 111.6 (d, *J* = 21.9 Hz) 115.3 (d, *J* = 3.5 Hz) 129.6, 129.7, 129.8, 130.0, 130.1, 133.6, 162.7 (d, *J* = 246.5 Hz) 164.9, 176.5. Analysis calculated for C_18_H_15_FN_2_O_3_ (326.33): C: 66.25, H: 4.63, N: 8.58; found C: 66.09, H: 4.39, N: 8.42.

##### 2-(2,5-Dioxopyrrolidin-1-yl)-*N*-(4-fluorophenyl)-2-phenylacetamide (**6**)

White solid, yield: 70.6%, (0.69 g); m.p. 143.4–143.9 °C; TLC: R_f_ = 0.48 (S_1_); UPLC (purity >99.9%): *t*_R_ = 5.59 min. LC–MS (ESI): *m*/*z* calculated for C_18_H_15_FN_2_O_3_ (M + H)^+^ 327.11, found 327.4. ^1^H-NMR (300 MHz, CDCl_3_) δ 2.75 (s, 4H, imide), 5.88 (s, 1H, CH), 6.92–6.99 (m, 2H, ArH), 7.30–7.38 (m, 2H, ArH), 7.40–7.46 (m, 3H, ArH), 7.48 (br. s, 1H, NH), 7.56–7.64 (m, 2H, ArH). ^13^C-NMR (75 MHz, CDCl_3_) δ 28.2, 59.1, 114.9–116.3, 122.1 (d, *J* = 8.1 Hz), 129.5, 129.7, 129.8, 133.0 (d, *J* = 3.5 Hz), 133.8, 159.6 (d, *J* = 244.2 Hz) 164.8, 176.6. Analysis calculated for C_18_H_15_FN_2_O_3_ (326.33): C: 66.25, H: 4.63, N: 8.58; found C: 66.31, H: 4.47, N: 8.62.

##### *N*-(2-Chlorophenyl)-2-(2,5-dioxopyrrolidin-1-yl)-2-phenylacetamide (**7**)

White solid, yield: 64.3%, (0.65 g); m.p. 76.8–78.6 °C; TLC: R_f_ = 0.56 (S_1_); UPLC (purity >99.9%): *t*_R_ = 6.19 min. LC–MS (ESI): *m*/*z* calculated for C_18_H_15_ClN_2_O_3_ (M + H)^+^ 343.08, found 343.8. ^1^H-NMR (300 MHz, CDCl_3_) δ 2.79 (s, 4H, imide), 5.90 (s, 1H, CH), 7.01 (td, *J* = 7.7, 1.4 Hz, 1H, ArH), 7.20–7.28 (m, 2H, ArH), 7.42–7.50 (m, 3H, ArH), 7.65–7.70 (m, 2H, ArH), 7.92 (br. s, 1H, NH), 8.34 (dd, *J* = 7.9, 1.4 Hz, 1H, ArH). ^13^C-NMR (75 MHz, CDCl_3_) δ 25.3, 28.3, 59.4, 121.2, 123.0, 125.2, 127.7, 128.9, 129.6, 129.8, 130.0, 133.6, 134.0, 164.8, 176.4. Analysis calculated for C_18_H_15_ClN_2_O_3_ (342.78): C: 63.07, H: 4.41, N: 8.17; found C: 63.31, H: 4.22, N: 8.25.

##### *N*-(3-Chlorophenyl)-2-(2,5-dioxopyrrolidin-1-yl)-2-phenylacetamide (**8**)

White solid, yield: 72.8%, (0.75 g); m.p. 109.4–109.9 °C; TLC: R_f_ = 0.49 (S_1_); UPLC (purity >99.9%): *t*_R_ = 6.26 min. LC–MS (ESI): *m*/*z* calculated for C_18_H_15_ClN_2_O_3_ (M + H)^+^ 343.08, found 343.8. ^1^H-NMR (300 MHz, CDCl_3_) δ 2.77 (s, 4H, imide), 5.89 (s, 1H, CH), 7.05–7.16 (m, 1H, ArH), 7.18–7.24 (m, 2H, ArH), 7.41–7.46 (m, 3H, ArH), 7.50–7.54 (m, 2H, NH, ArH), 7.57–7.62 (m, 2H, ArH). ^13^C-NMR (75 MHz, CDCl_3_) δ 28.2, 59.2, 118.1, 120.2, 125.0, 129.6, 129.8, 129.9, 133.5, 134.6, 138.1, 164.9, 176.5. Analysis calculated for C_18_H_15_ClN_2_O_3_ (342.78): C: 63.07, H: 4.41, N: 8.17; found C: 63.01, H: 4.69, N: 8.24.

##### *N*-(4-Chlorophenyl)-2-(2,5-dioxopyrrolidin-1-yl)-2-phenylacetamide (**9**)

White solid, yield: 69.6%, (0.72 g); m.p. 103.8–105.6 °C; TLC: R_f_ = 0.54 (S_1_); UPLC (purity >99.9%): *t*_R_ = 6.16 min. LC–MS (ESI): *m*/*z* calculated for C_18_H_15_ClN_2_O_3_ (M + H)^+^ 343.08, found 343.8. ^1^H-NMR (300 MHz, CDCl_3_) δ 2.76 (s, 4H, imide), 5.88 (s, 1H, CH), 7.20–7.24 (m, 1H, ArH), 7.25–7.35 (m, 3H, ArH), 7.40–7.45 (m, 3H, ArH), 7.52 (s, 1H, NH), 7.57–7.62 (m, 2H, ArH). ^13^C-NMR (75 MHz, CDCl_3_) δ 15.3, 28.2, 59.2, 65.8, 121.4, 129.0, 129.6, 129.7, 129.8, 130.0, 133.7, 135.6, 164.8, 176.5. Analysis calculated for C_18_H_15_ClN_2_O_3_ (342.78): C: 63.07, H: 4.41, N: 8.17; found C: 63.31, H: 4.34, N: 8.37.

##### (2-(2,5-Dioxopyrrolidin-1-yl)-2-phenyl-*N*-(2-(trifluoromethyl)phenyl)acetamide) (**10**)

White solid, yield: 71.1%, (0.80 g); m.p. 162.2–163.4 °C; TLC: R_f_ = 0.58 (S_1_); UPLC (purity > 99.9%): *t*_R_ = 6.59 min. LC–MS (ESI): *m*/*z* calculated for C_19_H_15_F_3_N_2_O_3_ (M + H)^+^ 377.10, found 377.5. ^1^H-NMR (300 MHz, CDCl_3_) δ 2.79 (s, 4H, imide), 5.98 (s, 1H, CH), 7.32–7.38 (m, 3H, ArH), 7.42–7.49 (m, 4H, ArH), 7.51–7.62 (m, 2H, ArH), 7.78 (s, 1H, NH). ^13^C-NMR (75 MHz, CDCl_3_) δ 27.8, 59.4, 116.2 (d, *J* = 271.8 Hz), 118.3, 124.7 (q, *J* = 32.3 Hz), 126.3 (q, *J* = 4.2 Hz) 128.2, 128.9, 129.4, 132.1, 140.3, 163.2, 177.4. Analysis calculated for C_19_H_15_F_3_N_2_O_3_ (376.34): C: 60.64, H: 4.02, N: 7.44; found C: 60.44, H: 4.42, N: 7.28.

##### 2-(2,5-Dioxopyrrolidin-1-yl)-2-phenyl-*N*-(3-(trifluoromethyl)phenyl)acetamide (**11**)

White solid, yield: 68.9%, (0.78 g); m.p. 158.2–159.4 °C; TLC: R_f_ = 0.60 (S_1_); UPLC (purity >99.9%): *t*_R_ = 6.52 min. LC–MS (ESI): *m*/*z* calculated for C_19_H_15_F_3_N_2_O_3_ (M + H)^+^ 377.10, found 377.5. ^1^H-NMR (300 MHz, CDCl_3_) δ 2.77 (s, 4H, imide), 5.92 (s, 1H, CH), 7.38 (d, *J* = 10.3 Hz, 1H, ArH), 7.37 (s, 1H, ArH), 7.41–7.47 (m, 3H, ArH), 7.55–7.66 (m, 4H, ArH), 7.70 (s, 1H, NH) ^13^C-NMR (75 MHz, CDCl_3_) δ 28.2, 59.2, 116.8, 116.8–116.9, 121.5 (q, *J* = 3.5 Hz), 123.7 (d, *J* = 272.9 Hz), 123.2, 129.5, 129.6, 129.8, 129.8, 131.4 (q, *J* = 33.0 Hz), 133.5, 137.5, 165.1, 176.5. Analysis calculated for C_19_H_15_F_3_N_2_O_3_ (376.34): C: 60.64, H: 4.02, N: 7.44; found C: 60.51, H: 4.27, N: 7.31.

##### 2-(2,5-Dioxopyrrolidin-1-yl)-2-phenyl-*N*-(4-(trifluoromethyl)phenyl)acetamide (**12**)

White solid, yield: 73.1%, (0.82 g); m.p. 188.1–188.5 °C; TLC: R_f_ = 0.58 (S_1_); UPLC (purity >99.9%): *t*_R_ = 6.59 min. LC–MS (ESI): *m*/*z* calculated for C_19_H_15_F_3_N_2_O_3_ (M + H)^+^ 377.10, found 377.5. ^1^H-NMR (300 MHz, CDCl_3_) δ 2.77 (s, 4H, imide), 5.92 (s, 1H, CH), 7.42–7.47 (m, 3H, ArH), 7.48–7.53 (m, 4H, ArH), 7.58–7.63 (m, 2H, ArH), 7.69 (s, 1H, NH). ^13^C-NMR (75 MHz, CDCl_3_) δ 28.2, 59.2, 118.5 (d, *J* = 271.8 Hz), 119.7, 126.1 (q, *J* = 32.3 Hz), 126.2 (q, *J* = 4.2 Hz) 129.6, 129.7, 129.8, 133.5, 140.0, 165.1, 176.5. Analysis calculated for C_19_H_15_F_3_N_2_O_3_ (376.34): C: 60.64, H: 4.02, N: 7.44; found C: 60.52, H: 4.34, N: 7.31.

##### 2-(2,5-Dioxopyrrolidin-1-yl)-2-phenyl-*N*-(3-(trifluoromethoxy)phenyl)acetamide (**13**)

White solid, yield: 68.7%, (0.79 g); m.p. 132.6–133.4 °C; TLC: R_f_ = 0.66 (S_2_); UPLC (purity >99.9%): *t*_R_ = 6.76 min. LC–MS (ESI): *m*/*z* calculated for C_19_H_15_F_3_N_2_O_4_ (M + H)^+^ 393.10, found 393.2. ^1^H-NMR (500 MHz, CDCl_3_) δ 2.77 (s, 4H, imide), 5.88 (s, 1H, CH), 5.98 (br. s, 1H, NH), 7.09–7.12 (m, 2H, ArH), 7.18 (d, *J* = 8.0 Hz, 1H, ArH), 7.33–7.43 (m, 4H, ArH), 7.59–7.62 (m, 2H, ArH). ^13^C-NMR (126 MHz, CDCl_3_) δ 27.5, 56.2, 118.2, 120.2, 120.8 (q, *J* = 257.1 Hz), 124.3, 127.2, 128.8, 129.9, 130.8, 132.4, 141.7, 147.2, 166.2, 175.7. Analysis calculated for C_19_H_15_F_3_N_2_O_4_ (392.33): C: 58.17, H: 3.85, N: 7.14, found C: 58.43, H: 3.98, N: 7.03.

##### *N*-Benzyl-2-(2,5-dioxopyrrolidin-1-yl)-2-phenylacetamide (**14**)

White solid, yield: 71.2%, (0.69 g); m.p. 128.3–129.9 °C; TLC: R_f_ = 0.45 (S_1_); UPLC (purity >99%): *t*_R_ = 5.24 min. LC–MS (ESI): *m*/*z* calculated for C_19_H_18_N_2_O_3_ (M + H)^+^ 323.13, found 323.3. ^1^H-NMR (300 MHz, CDCl_3_): δ 2.77 (s, 4H, imide), 4.35–4.44 (m, 1H, CH_2_), 4.51–4.61 (m, 1H, CH_2_), 5.77(s, 1H, CH), 5.96 (br. s, 1H, NH), 7.22–7.40 (m, 5H, ArH), 7.54–7.64 (m, 5H, ArH). ^13^C-NMR (75 MHz, CDCl_3_): δ 28.2, 43.9, 58.5, 76.8, 77.5, 127.1, 127.5, 128.4, 130.3, 134.1, 137.6, 166.5, 176.4. Analysis calculated for C_19_H_18_N_2_O_3_ (322.36): C: 70.79, H: 5.63, N: 8.69; found C: 70.89, H: 5.61, N: 8.72.

##### 2-(2,5-Dioxopyrrolidin-1-yl)-*N*-(2-fluorobenzyl)-2-phenylacetamide (**15**)

White solid, yield: 74.5%, (0.76 g); mp. 148.3–149.3 °C; TLC: R_f_ = 0.55 (S_1_); UPLC (purity >99.9%): *t*_R_ = 5.36 min. LC–MS (ESI): *m*/*z* calculated for C_19_H_17_N_2_O_3_F (M + H)^+^ 341.12, found 341.13. ^1^H-NMR (300 MHz, CDCl_3_): δ 2.75 (s, 4H, imide), 4.50 (dd, *J* = 5.7, 4.1 Hz, 2H, CH_2_), 5.74 (s, 1H, CH), 6.02 (br. s, 1H, NH), 6.94–7.03 (m, 1H, ArH), 7.07–7.15 (m, 1H, ArH), 7.19–7.28 (m, 1H, ArH), 7.33–7.41 (m, 4H, ArH), 7.54–7.59 (m, 2H, ArH). ^13^C-NMR (75 MHz, CDCl_3_): δ 28.2, 38.0 (d, *J* = 4.6 Hz), 58.5, 115.1, 115.4, 124.3, 124.5, 124.7, 129.3 (d, *J* = 8.1 Hz), 129.4, 129.5, 129.9, 134.1, 160.7 (dd, *J* = 249.9, 1.0 Hz), 166.7, 176.5. Analysis calculated for C_19_H_17_N_2_O_3_F (340.35): C: 67.05, H: 5.03, N: 5.58; found C: 67.01, H: 5.36, N: 5.29.

##### 2-(2,5-Dioxopyrrolidin-1-yl)-*N*-(3-fluorobenzyl)-2-phenylacetamide (**16**)

White solid, yield: 69.4%, (0.71 g); m.p. 136.7–137.7 °C; TLC: R_f_ = 0.49 (S_1_); UPLC (purity >99.9%): *t*_R_ = 5.40 min. LC–MS (ESI): *m*/*z* calculated for C_19_H_17_N_2_O_3_F (M + H)^+^ 341.12, found 341.3. ^1^H-NMR (300 MHz, CDCl_3_): δ 2.77 (s, 4H, imide) 4.52 (dd, *J* = 15.4, 5.6 Hz, 2H, CH_2_), 5.76 (s, 1H, CH), 5.98 (br. s, 1H, NH), 6.90–7.05 (m, 3H, ArH), 7.23–7.32 (m, 1H, ArH), 7.35–7.42 (m, 3H, ArH), 7.56–7.61 (m, 2H, ArH). ^13^C-NMR (75 MHz, CDCl_3_): δ 28.2, 43.4, 58.5, 114.3 (d, *J* = 23.0 Hz), 114.4 (d, *J* = 20.7 Hz), 122.8, 129.7 (d, *J* = 28.8 Hz), 129.5, 130.2 (d, *J* = 9.2 Hz), 134.1, 140.3 (d, *J* = 6.9 Hz), 163.0 (d, *J* = 246.5 Hz), 166.8, 176.5. Analysis calculated for C_19_H_17_N_2_O_3_F (340.35): C: 67.05, H: 5.03, N: 5.58; found C: 67.19, H: 5.22, N: 5.66.

##### 2-(2,5-Dioxopyrrolidin-1-yl)-*N*-(4-fluorobenzyl)-2-phenylacetamide (**17**)

White solid, yield: 65.7%, (0.67 g); mp. 146.3–147.7 °C; TLC: R_f_ = 0.45 (S_1_); UPLC (purity >99,9%): *t*_R_ = 5.39 min. LC–MS (ESI): *m*/*z* calculated for C_19_H_17_N_2_O_3_F (M + H)^+^ 341.12, found 341.1. ^1^H-NMR (300 MHz, CDCl_3_): δ 2.76 (s, 4H, imide), 4.31 (dd, *J* = 15.0, 5.5 Hz, 2H, CH_2_), 5.75 (s, 1H, CH), 5.95 (br. s, 1H, NH), 6.95–7.03 (m, 2H, ArH), 7.18–7.27 (m, 2H, ArH), 7.34–7.40 (m, 3H, ArH), 7.52–7.59 (m, 2H, ArH). ^13^C-NMR (75 MHz, CDCl_3_): δ 28.2, 43.2, 58.5, 115.5 (d, *J* = 21.9 Hz), 129.1 (d, *J* = 8.1 Hz), 129.4, 129.9, 133.5 (d, *J* = 3.5 Hz), 134.2, 162.1 (d, *J* = 246.5 Hz), 166.6, 176.5. Analysis calculated for C_19_H_17_N_2_O_3_F (340.35): C: 67.05, H: 5.03, N: 5.58; found C: 67.18, H: 5.29, N: 5.41.

##### *N*-(2-Chlorobenzyl)-2-(2,5-dioxopyrrolidin-1-yl)-2-phenylacetamide (**18**)

White solid, yield: 66.3%, (0.71 g); m.p. 152.0–153.6 °C; TLC: R_f_ = 0.55 (S_1_); UPLC (purity 97%): *t*_R_ = 5.79 min. LC–MS (ESI): *m*/*z* calculated for C_19_H_17_N_2_O_3_Cl (M + H)^+^ 357.09, found 357.1. ^1^H-NMR (300 MHz, CDCl_3_): δ 2.75 (s, 4H, imide), 4.53 (dd, *J* = 6.1, 2.4 Hz, 2H, CH_2_), 5.74 (s, 1H, CH), 6.11 (br. s, 1H, NH), 7.16–7.28 (m, 2H, ArH), 7.29–7.34 (m, 1H, ArH), 7.35–7.43 (m, 4H, ArH), 7.54–7.60 (m, 2H, ArH). ^13^C-NMR (75 MHz, CDCl_3_): δ 28.2, 42.0, 58.5, 76.4, 78.1, 127.1, 128.4, 130.0, 133.3, 134.1, 134.9, 166.6, 176.4. Analysis calculated for C_19_H_17_N_2_O_3_Cl (356.81): C: 63.96, H: 4.80, N: 7.85; found C: 63.91, H: 4.65, N: 7.92.

##### *N*-(3-Chlorobenzyl)-2-(2,5-dioxopyrrolidin-1-yl)-2-phenylacetamide (**19**)

White solid, yield: 79.2%, (0.85 g); m.p. 137.3–138.8 °C; TLC: R_f_ = 0.7 (S_1_); UPLC (purity 99.5%): *t*_R_ = 5.87 min. LC–MS (ESI): *m*/*z* calculated for C_19_H_17_N_2_O_3_Cl (M + H)^+^ 357.09, found 357.1. ^1^H-NMR (300 MHz, CDCl_3_): δ 2.76 (s, 4H, imide), 4.28–4.58 (m, 2H, CH_2_), 5.76 (s, 1H, CH), 5.99 (br. s, 1H, NH), 7.12–7.18 (m, 1H, ArH), 7.20–7.28 (m, 3H, ArH), 7.35–7.41 (m, 3H, ArH), 7.55–7.61 (m, 2H, ArH). ^13^C-NMR (75 MHz, CDCl_3_): δ 28.2, 43.3, 58.5, 77.3–78.1, 125.5, 127.5 (d, *J* = 9.2 Hz), 129.3–130.4, 133.8–134.6, 139.7, 166.7, 176.4. Analysis calculated for C_19_H_17_N_2_O_3_Cl (356.81): C: 63.96, H: 4.80, N: 7.85; found C: 63.88, H: 4.62, N: 7.81.

##### *N*-(4-Chlorobenzyl)-2-(2,5-dioxopyrrolidin-1-yl)-2-phenylacetamide (**20**)

White solid, yield: 75.7%, (0.81 g); m.p. 146.3–147.9 °C; TLC: R_f_ = 0.7 (S_1_); UPLC (purity 96.5%): *t*_R_ = 5.91 min. LC–MS (ESI): *m*/*z* calculated for C_19_H_17_N_2_O_3_Cl (M + H)^+^ 357.09, found 357. ^1^H-NMR (300 MHz, CDCl_3_): δ 2.76 (s, 4H, imide), 4.30 (dd, *J* = 15.2, 5.4 Hz, 1H, CH_2_), 4.55 (dd, *J* = 15.2, 6.6 Hz, 1H, CH_2_), 5.75 (s, 1H, CH), 5.95 (br. s, 1H, NH), 7.16–7.22 (m, 2H, ArH), 7.25–7.30 (m, 2H, ArH), 7.34–7.40 (m, 3H, ArH), 7.54–7.59 (m, 2 H, ArH). ^13^C-NMR (75 MHz, CDCl_3_): δ 28.2, 43.2, 58.5, 77.3–78.1, 128.8 (d, *J* = 2.3 Hz), 129.2–130.0, 133.3, 134.1, 136.2, 166.6, 176.4. Analysis calculated for C_19_H_17_N_2_O_3_Cl (356.81): C: 63.96, H: 4.80, N: 7.85; found C: 63.81, H: 4.92, N: 7.67.

##### 2-(2,5-Dioxopyrrolidin-1-yl)-2-phenyl-*N*-(2-(trifluoromethyl)benzyl)acetamide (**21**)

White solid, yield: 67.1%, (0.78 g); m.p. 137.7–139.1 °C; TLC: R_f_ = 0.58 (S_2_); UPLC (purity 97%): *t*_R_ = 6.17 min. LC–MS (ESI): *m*/*z* calculated for C_20_H_17_N_2_O_3_F_3_ (M + H)^+^ 391.12, found 391.1. ^1^H-NMR (300 MHz, CDCl_3_): δ 2.76 (s, 4H, imide) 4.60 (dd, *J* = 5.9 Hz, 1H, CH_2_) 4.66 (dd, *J* = 6.7 Hz, 1H, CH_2_) 5.74 (s, 1H, CH) 6.01 (br. s, 1H, NH) 7.33–7.42 (m, 4H, ArH) 7.51–7.63 (m, 5H, ArH). ^13^C-NMR (75 MHz, CDCl_3_): δ 28.2, 40.7 (d, *J* = 2.3 Hz), 58.5, 124.2 (d, *J* = 249.9 Hz), 125.9 (d, *J* = 5.8 Hz), 126.1 (d, *J* = 5.8 Hz), 127.6, 129.4, 129.5, 129.8, 130.2, 132.4, 134.0, 166.8, 176.5. Analysis calculated for C_20_H_17_N_2_O_3_F_3_ (390.36): C: 61.54, H: 4.39, N: 7.18; found C: 61.47, H: 4.21, N: 7.25.

##### 2-(2,5-Dioxopyrrolidin-1-yl)-2-phenyl-*N*-(3-(trifluoromethyl)benzyl)acetamide (**22**)

White solid, yield: 71.4%, (0.84); m.p. 152.8–153.4 °C; TLC: R_f_ = 0.54 (S_2_); UPLC (purity 98%): *t*_R_ = 6.19 min. LC–MS (ESI): *m*/*z* calculated for C_20_H_17_N_2_O_3_F_3_ (M + H)^+^ 391.12, found 391.0. ^1^H-NMR (300 MHz, CDCl_3_): δ 2.78 (s, 4H, imide) 4.39 (dd, *J* = 15.4, 5.4 Hz, 1H, CH_2_) 4.65 (dd, *J* = 15.4, 6.7 Hz, 1H, CH_2_) 5.78 (s, 1H, CH) 6.02 (br. s, 1H, NH) 7.35–7.41 (m, 1H, ArH) 7.42–7.51 (m, 4H, ArH) 7.52–7.63 (m, 4H, ArH). ^13^C-NMR (75 MHz, CDCl_3_): δ 28.2, 43.4, 58.5, 124.0 (d, *J* = 272.9 Hz), 124.1 (q, *J* = 3.8 Hz) 124.3 (q, *J* = 3.5 Hz), 129.4 (d, *J* = 27.6 Hz), 129.7 (d, *J* = 25.3 Hz), 132.4 (d, *J* = 249.9 Hz), 138.8, 166.9, 176.5. Analysis calculated for C_20_H_17_N_2_O_3_F_3_ (390.36): C: 61.54, H: 4.39, N:7.18; found C: 61.32, H: 4.48, N: 7.02.

##### 2-(2,5-Dioxopyrrolidin-1-yl)-2-phenyl-*N*-(4-(trifluoromethyl)benzyl)acetamide (**23**)

White solid, yield: 62.9%, (0.74 g); m.p. 174.5–176.0 °C; TLC: R_f_ = 0.86 (S_2_); UPLC (purity 98%): *t*_R_ = 6.25 min. LC–MS (ESI): *m*/*z* calculated for C_20_H_17_N_2_O_3_F_3_ (M + H)^+^ 391.12, found 391.1. ^1^H-NMR (300 MHz, CDCl_3_): δ 2.77 (s, 4H, imide), 4.37 (dd, *J* = 15.6, 5.4 Hz, 1H, CH_2_), 4.66 (dd, *J* = 15.6, 6.8 Hz, 1H, CH_2_), 5.77 (s, 1H, CH) 6.03 (br. s, 1H, NH), 7.35–7.44 (m, 5H, ArH), 7.55–7.61 (m, 4H, ArH). ^13^C-NMR (75 MHz, CDCl_3_): δ 28.2, 43.4, 58.5, 124.1 (d, *J* = 271.8 Hz), 125.6 (d, *J* = 11.5 Hz), 125.6 (d, *J* = 4.6 Hz), 127.6, 129.5, 129.8, 134.1, 141.8, 166.9,176.5. Analysis calculated for C_20_H_17_N_2_O_3_F_3_ (390.36): C: 61.54, H: 4.39, N: 7.18; found C: 61.49, H: 4.21, N: 7.24.

##### 2-(2,5-Dioxopyrrolidin-1-yl)-2-phenyl-*N*-(3-(trifluoromethoxy)benzyl)acetamide (**24**)

White solid, yield: 67.4%, (0.82 g); m.p. 129.6–130.4 °C; TLC: R_f_ = 0.64 (S_2_); UPLC (purity >99.9%): *t*_R_ = 6.41 min. LC–MS (ESI): *m*/*z* calculated for C_20_H_17_F_3_N_2_O_4_ (M + H)^+^ 407.11, found 407.2. ^1^H-NMR (500 MHz, CDCl_3_) δ 2.75 (s, 4H, imide), 4.34 (dd, *J* = 15.5, 5.7 Hz, 1H, CH_2_), 4.59 (dd, *J* = 15.5, 6.9 Hz, 1H, CH_2_), 5.75 (s, 1H, CH), 6.00 (br. s, 1H, NH), 7.07–7.11 (m, 2H, ArH), 7.20 (d, *J* = 8.0 Hz, 1H, ArH), 7.31–7.39 (m, 4H, ArH), 7.56–7.58 (m, 2H, ArH). ^13^C-NMR (126 MHz, CDCl_3_) δ 28.3, 43.4, 58.6, 120.5 (q, *J* = 257.1 Hz), 119.9, 120.0, 125.8, 129.6, 129.6, 129.9, 130.2, 134.2, 140.2, 149.5, 166.9, 176.6. Analysis calculated for C_20_H_17_F_3_N_2_O_4_ (406.36): C: 59.11, H: 4.22, N: 6.89; found C: 59.19, H: 4.38, N: 6.95.

##### 2-(2,5-Dioxopyrrolidin-1-yl)-*N*-phenethyl-2-phenylacetamide (**25**)

White solid, yield: 73.5% (0.74 g); m.p. 138.4–139.2 °C; TLC: R_f_ = 0.4 (S_2_); UPLC (purity 99.0%): *t*_R_ = 5.57 min. LC–MS (ESI): *m*/*z* calculated for C_20_H_20_N_2_O_3_ (M + H)^+^ 337.15, found 337.2. ^1^H-NMR (500 MHz, CDCl_3_) δ 2.73 (s, 4H, imide), 2.76 (t, *J* = 6.8 Hz, 2H, CH_2_), 3.34–3.41 (m, 1H, CH_2_), 3.60 (dd, *J* = 13.4, 6.6 Hz, 1H, CH_2_), 5.59 (br. s, 1H, NH), 5.63 (s, 1H, CH), 7.07–7.10 (m, 2H, ArH), 7.15–7.20 (m, 1H, ArH), 7.20–7.29 (m, 3H, ArH), 7.30–7.33 (m, 2H, ArH), 7.44 (d, *J* = 6.7 Hz, 2H, ArH). ^13^C-NMR (126 MHz, CDCl_3_) δ 28.3, 35.3, 41.4, 58.7, 126.6, 128.6, 128.7, 128.8, 128.9, 129.4, 129.8, 134.3, 138.6, 166.6, 176.6. Analysis calculated for C_20_H_20_N_2_O_3_ (336.39): C: 71.41, H: 5.99, N: 8.33; found C: 71.29, H: 5.87, N: 8.49.

##### 2-(2,5-Dioxopyrrolidin-1-yl)-*N*-(2-fluorophenethyl)-2-phenylacetamide (**26**)

White solid, yield: 69.8% (0.73 g); m.p. 142.4–142.8 °C; TLC: R_f_ = 0.44 (S_2_); UPLC (purity >99.9%): *t*_R_ = 5.66 min. LC–MS (ESI): *m*/*z* calculated for C_20_H_19_FN_2_O_3_ (M + H)^+^ 355.14, found 355.2. ^1^H-NMR (500 MHz, CDCl_3_) δ 2.74 (s, 4H, imide), 2.77–2.81 (m, 1H, CH_2_), 2.82–2.87 (m, 1H, CH_2_), 3.42 (dd, *J* = 13.7, 6.8 Hz, 1H, CH_2_), 3.56 (dd, *J* = 13.4, 6.5 Hz, 1H, CH_2_), 5.63 (s, 2H, NH, CH), 6.95 (t, *J* = 8.8 Hz, 1H, ArH), 7.02 (t, *J* = 7.3 Hz, 1H, ArH), 7.13–7.18 (m, 2H, ArH), 7.30–7.34 (m, 3H, ArH), 7.46 (d, *J* = 6.2 Hz, 2H, ArH). ^13^C-NMR (126 MHz, CDCl_3_) δ 28.3, 28.9, 40.2, 58.7, 115.4 (d, *J* = 22.3 Hz), 124.2 (d, *J* = 3.6 Hz), 125.5 (d, *J* = 15.7 Hz), 128.4 (d, *J* = 8.4 Hz), 129.4, 129.4, 129.9, 131.4 (d, *J* = 4.8 Hz), 134.2, 161.2 (d, *J* = 245.1 Hz), 166.7, 176.6. Analysis calculated for C_20_H_19_FN_2_O_3_ (354.38): C: 67.79, H: 5.40, N: 7.91; found C: 67.61, H: 5.51, N: 7.84.

##### *N*-(3-Chlorophenethyl)-2-(2,5-dioxopyrrolidin-1-yl)-2-phenylacetamide (**27**)

White solid, yield: 73.4% (0.82 g); mp. 137.4–138.2 °C; TLC: R_f_ = 0.62 (S_2_); UPLC (purity >99.9%): *t*_R_ = 6.20 min. LC–MS (ESI): *m*/*z* calculated for C_20_H_19_ClN_2_O_3_ (M + H)^+^ 371.11, found 371.4. ^1^H-NMR (500 MHz, CDCl_3_) δ 2.73–2.77 (m, 6H, imide, CH_2_), 3.29 (td, *J* = 13.3, 6.6 Hz, 1H, CH_2_), 3.63 (dd, *J* = 13.5, 6.6 Hz, 1H, CH_2_), 5.57 (t, *J* = 5.2 Hz, 1H, NH), 5.63 (s, 1H, CH), 6.99–7.01 (m, 1H, ArH), 7.14–7.17 (m, 3H, ArH), 7.32–7.34 (m, 3H, ArH), 7.44–7.47 (m, 2H, ArH). ^13^C-NMR (126 MHz, CDCl_3_) δ 28.3, 35.1, 41.2, 58.7, 126.8, 127.1, 129.2, 129.4, 129.5, 129.7, 129.9, 134.2, 134.4, 140.8, 166.7, 176.6. Analysis calculated for C_20_H_19_ClN_2_O_3_ (370.83): C: 64.78, H: 5.16, N: 7.55; found C: 64.99, H: 5.05, N: 7.81.

##### *N*-(4-Chlorophenethyl)-2-(2,5-dioxopyrrolidin-1-yl)-2-phenylacetamide (**28**)

White solid, yield: 68.6% (0.76 g); m.p. 137.2–137.8 °C; TLC: R_f_ = 0.52 (S_2_); UPLC (purity >99.9%): *t*_R_ = 6.10 min. LC–MS (ESI): *m*/*z* calculated for C_20_H_19_ClN_2_O_3_ (M + H)^+^ 371.11, found 371.4. ^1^H-NMR (500 MHz, CDCl_3_) δ 2.72–2.76 (m, 6H, imide, CH_2_), 3.31 (dd, *J* = 13.2, 5.7 Hz, 1H, CH_2_), 3.59 (dd, *J* = 13.2, 6.3 Hz, 1H, CH_2_), 5.56 (t, *J* = 5.2 Hz, 1H, NH), 5.62 (s, 1H, CH), 7.02–7.05 (m, 2H, Ar), 7.18–7.20 (m, 2H, ArH), 7.29–7.35 (m, 3H, ArH), 7.42 (d, *J* = 6.8 Hz, 2H, ArH). ^13^C-NMR (126 MHz, CDCl_3_) δ 28.3, 34.7, 41.2, 58.7, 128.8, 129.4, 129.4, 129.8, 130.2, 132.4, 134.2, 137.1, 166.6, 176.6. Analysis calculated for C_20_H_19_ClN_2_O_3_ (370.83): C: 64.78, H: 5.16, N: 7.55; found C: 64.53, H: 5.39, N: 7.72.

##### 2-(2,5-Dioxopyrrolidin-1-yl)-2-phenyl-*N*-(3-(trifluoromethyl)phenethyl)acetamide (**29**)

White solid, yield: 69.3% (0.80 g); m.p. 149.2–150.4 °C; TLC: R_f_ = 0.58 (S_2_); UPLC (purity >99.9%): *t*_R_ = 6.92 min. LC–MS (ESI): *m*/*z* calculated for C_21_H_19_F_3_N_2_O_3_ (M + H)^+^ 405.14, found 405.3. ^1^H-NMR (500 MHz, CDCl_3_) δ 2.75 (s, 4 H, imide) 2.81–2.89 (m, 2 H, CH_2_) 3.29–3.38 (m, 1 H, CH_2_) 3.66 (dd, *J* = 13.5, 6.6 Hz, 1 H, CH_2_) 5.59 (br. s, 1 H, NH) 5.63 (s, 1 H, CH) 7.28–7.33 (m, 3 H, ArH) 7.33–7.39 (m, 2 H, ArH) 7.41–7.47 (m, 4 H, ArH). ^13^C-NMR (126 MHz, CDCl_3_) δ 28.3, 35.2, 41.3, 58.7, 124.15 (q, *J* = 272.2 Hz), 123.5 (q, *J* = 3.6 Hz), 125.7 (q, *J* = 3.6 Hz), 129.1, 129.4, 129.5, 129.7, 130.1 (q, *J* = 32.0 Hz), 132.4, 134.2, 139.8, 166.8 176.6. Analysis calculated for C_21_H_19_F_3_N_2_O_3_ (404.39): C: 62.37, H: 4.74, N: 6.93, found C: 62.22, H: 4.66, N: 7.02.

##### 2-(2,5-Dioxopyrrolidin-1-yl)-2-phenyl-*N*-(3-(trifluoromethoxy)phenethyl)acetamide (**30**)

White solid, yield: 64.3% (0.81 g); m.p. 113.3–114.2 °C; TLC: R_f_ = 0.62 (S_2_); UPLC (purity >99.9%): *t*_R_ = 6.64 min. LC–MS (ESI): *m*/*z* calculated for C_21_H_19_F_3_N_2_O_4_ (M + H)^+^ 421.13, found 421.1. ^1^H-NMR (500 MHz, CDCl_3_) δ 2.75 (s, 4H), 2.80 (t, *J* = 6.9 Hz, 2H, CH_2_), 3.30–3.38 (m, 1H, CH_2_), 3.63 (dd, *J* = 13.5, 6.6 Hz, 1H, CH_2_), 5.59 (t, *J* = 5.4 Hz, 1H, NH), 5.64 (s, 1H, CH), 6.99 (s, 1H, ArH), 7.03–7.07 (m, 2H, ArH), 7.25–7.34 (m, 4H, ArH), 7.45 (d, *J* = 6.6 Hz, 2H, ArH). ^13^C-NMR (126 MHz, CDCl_3_) δ 28.3, 35.1, 41.2, 58.7, 120.2 (d, *J* = 309.6 Hz), 120.5 (d, *J* = 256.5 Hz), 127.3, 129.4, 129.7 (t, *J* = 31.1 Hz), 134.2, 141.1, 149.5, 166.7, 176.6. Analysis calculated for C_21_H_19_F_3_N_2_O_4_ (420.39): C: 60.00, H: 4.56, N: 6.66; found C: 60.21, H: 4.42, N: 6.48.

##### 2-(2,5-Dioxopyrrolidin-1-yl)-2-phenyl-*N*-(3-phenylpropyl)acetamide (**31**)

White solid, yield: 73.7% (0.77 g); m.p. 133.1–133.9 °C; TLC: R_f_ = 0.50 (S_2_); UPLC (purity >99.9%): *t*_R_ = 6.16 min. LC–MS (ESI): *m*/*z* calculated for C_21_H_22_N_2_O_3_ (M + H)^+^ 351.16, found 351.2. ^1^H-NMR (500 MHz, CDCl_3_) δ 1.78 (td, *J* = 7.5, 2.9 Hz, 2H, CH_2_), 2.58 (t, *J* = 7.7 Hz, 2H, CH_2_), 2.74 (s, 4H, imide), 3.18–3.22 (m, 1H, CH_2_), 3.31–3.36 (m, 1H, CH_2_), 5.55–5.63 (m, 1H, CH_2_), 5.66 (s, 1H, NH), 7.09–7.17 (m, 3H, ArH), 7.22–7.24 (m, 2H, ArH), 7.38–7.40 (m, 3H, ArH), 7.53–7.55 (m, 2H, ArH). ^13^C-NMR (126 MHz, CDCl_3_) δ 28.3, 31.0, 33.1, 39.7, 58.7, 126.1, 128.4, 128.5, 129.5, 129.9, 134.5, 141.3, 166.6, 176.6. Analysis calculated for C_21_H_22_N_2_O_3_ (350.42): C: 71.98, H: 6.33, N: 7.99; found C: 71.62, H: 6.28, N: 8.12.

### 3.2. Anticonvulsant Activity

Experiments were performed on adult male CD-1 mice weighing 22–26 g, purchased from the Animal House at the Faculty of Pharmacy, Jagiellonian University Medical College, Cracow, Poland. The animals were kept at room temperature of 20 ± 2 °C under standard conditions. Initial qualitative efficacy screening was conducted in groups of four mice. To obtain the ED_50_ (median effective dose), at least three groups of six mice were injected with various doses of tested compounds. The anticonvulsant activity studies were approved by the Local Ethical Committee in Cracow, Poland (No 111/2016, 165/2018, 228A/2019, 360/2019) and conducted in compliance with the European Union Directive of 22 September 2010 (2010/63/EU). The tested substances were suspended in a 1% solution of Tween-80 and administered via the intraperitoneal (i.p.) route in a volume of 10 mL/kg body weight.

LTG, and VPA were purchased in Sigma-Aldrich (St. Louis, MO, USA), LCS in UCB Pharma (Braine-l’Alleud, Belgium). The pretreatment times for the aforementioned reference AEDs were based on information from the National Institute of Neurological Disorders and Stroke (NINDS) Epilepsy Therapy Screening Program (ETSP) [38]. The in vivo procedures for the following tests are reported elsewhere: maximal electroshock seizure test (MES) [14], the 6 Hz (32 mA) seizure model [14], and the rotarod test for acute neurological toxicity [9].

### 3.3. Antinociceptive Activity

The experimental groups consisted of 6–12 adult male Albino Swiss mice (CD-1, 18–25 g). Each animal was tested only once. Immediately after the assay, the animals were sacrificed by cervical dislocation. Behavioral measurements were observed by trained observers. The in vivo antinociceptive assays were in accordance with Polish regulations and European Union Directive of 22 September 2010 (2010/63/EU). All procedures were carried out according to the rules of the International Council on Laboratory Animal Science (ICLAS) and were approved by the Local Ethical Committee in Cracow, Poland (approval No 104/2015, 111/2016, and 179/2017). The tested substances were suspended in 1% aqueous solution of Tween-80 and were injected i.p. 30 min prior to the test. Control group animals (negative control) were administered with an appropriate amount of vehicle (Tween-80, 1% aqueous solution, i.p.) 30 min prior to the test.

The experimental in vivo procedures were previously reported for the formalin test [39]; compounds **4** and **30** were tested in three doses—40, 80, 120 mg/kg (**4**), and 20, 40, 80 mg/kg (**30**). Before formalin application, different groups of animals were injected i.p. with vehicle (10 mL/kg, negative control). The in vivo procedure for the model of capsaicin-induced nociception was previously reported [40]; the animals were pretreated with vehicle (10 mL/kg, negative control) and the dose–response of investigated compounds was evaluated at 40, 80, 120 mg/kg (**4**), and 20, 40, 80 mg/kg (**30**). The in vivo procedure for the model of OXPT-induced peripheral neuropathy was previously reported [41]; the mice with developed tactile allodynia were pretreated i.p. with test compound **30** (20, 40, and 80 mg/kg) and vehicle.

### 3.4. In Vivo Data Analysis

#### 3.4.1. Anticonvulsant Activity and Neurotoxicity Studies

The probit analysis [23] was used to calculate the ED_50_ and TD_50_ values with 95% confidence limits. The PI values (protective indexes) for the tested substance and standard AEDs were calculated by dividing the TD_50_ value, as determined in the rotarod test, by the respective ED_50_ value, as determined in the MES or 6 Hz (32 mA) tests.

#### 3.4.2. Antinociceptive Activity Studies

Data are presented as means ± standard error of the mean (SEM). The GraphPad Prism Software (v.5) was used to analyze the vast majority of data. Statistically significant differences between groups were calculated using one-way analysis of variance (ANOVA) and the post hoc Dunnett’s multiple comparison test or two-way analysis of variance (ANOVA). The criterion for significance was set at *p* < 0.05. The log-probit method was applied to statistically determine the ED_50_ values with 95% confidence limits.

### 3.5. In Vitro ADME-Tox Studies

#### 3.5.1. Permeability

Precoated PAMPA Plate System Gentest™ was provided by Corning, (Tewksbury, MA, USA). The detailed procedure and proper formulas were described previously [36].

#### 3.5.2. Metabolic Stability

These assays were performed on human liver microsomes (HLMs), purchased from Sigma-Aldrich (St. Louis, MO, USA), according to [14].

#### 3.5.3. Influence on Recombinant Human CYP3A4, CYP2D6, and CYP2C9 Cytochrome P450 Isoforms

The luminescent CYP3A4 P450-Glo™, CYP2D6 P450-Glo™, and CYP2C9 P450-Glo™ assays and protocols were provided by Promega (Madison, WI, USA) [42]. The detailed procedures are reported in the literature (CYP3A4 [14,15], CYP2D6 [15], and CYP2C9 [36]).

#### 3.5.4. Hepatotoxicity Assessment

These studies were performed on hepatoma HepG2 cells, according to a protocol reported previously [43]

### 3.6. In Silico Studies

The biotransformation pathways for metabolites 2, 24, and 30 were estimated in silico by MetaSite 6.0.1 provided by Molecular Discovery Ltd. (Hertfordshire, UK). The metabolic pathways and probable metabolite structures were studied using a liver computational model [44].

### 3.7. In Vitro Binding and Functional Assays

Binding studies were carried out commercially in Eurofines Laboratories (Poitiers, France). The functional assays were performed in Eurofins Panlabs Discovery Services Taiwan, Ltd. (New Taipei City, Taiwan). All procedures are described elsewhere (for details, see Table S4 in Supplementary Materials).

## 4. Conclusions

In the current studies, we developed a new series of hybrid anticonvulsants with an incorporated phenylglycine moiety. These compounds were designed as analogues or previously reported alanine derivatives which showed broad-spectrum and potent anticonvulsant efficacy. As a result, several compounds described herein revealed potent protection in the maximal electroshock (MES) test and the psychomotor 6 Hz (32 mA) seizure model in mice, which are recognized as the standard and widely applied seizure models for early identification of new AED candidates. The most potent anticonvulsant activity and satisfactory safety profile were demonstrated for compound **30**. In addition, **30** was effective in the formalin test of tonic pain, the capsaicin-induced pain model, and the oxaliplatin-induced neuropathic pain model in mice. The plausible mechanism of action of **30** is probably related to the inhibition of calcium currents mediated by Cav_1.2_ (L-type) channels. Notably, **30** revealed high metabolic stability on human liver microsomes, negligible hepatotoxicity, and relatively weak inhibition of cytochrome P450 isoenzymes. In conclusion, compound **30** seems to be worthy of further and more detailed characterization as a potential candidate for the treatment of epilepsy and neuropathic pain.

## Figures and Tables

**Table 1:** The quantitative pharmacological parameters: median effective dose (ED_50_), median toxic dose (TD_50_), and protective indexes (PIs) in mice intraperitoneally (i.p.) injected.

Compound	TPE (h) *^a^*	ED_50_ MES (mg/kg) *^b^*	ED_50_ 6 Hz (32 mA) (mg/kg) *^c^*	TD_50_ (rotarod) (mg/kg) *^d^*	PI (TD_50_/ED_50_) *^e^*
**3**	0.5	>130	92.5 (72.3–118.3)	>300	>3.2 (6 Hz)
**4**	0.5	73.2 (58.3–92.8)	–	>300	>4.1 (MES)
**6**	0.5	117.7 (92.6–149.6)	57.6 (33.9–97.9)	>300	>2.5 (MES) >5.2 (6 Hz)
**8**	0.5	73.5 (59.3–90.4)	–	>300	>4.1 (MES)
**9**	0.5	105.8 (100.3–111.6)	–	>300	>2.8 (MES)
**14**	0.5	>130	62.9 (45.7–86.7)	>300	>4.7 (6 Hz)
**19**	0.5	90.7 (82.6–99.5)	89.9 (74.4–108.5)	>300	>3.3 (MES) >3.3 (6 Hz)
**21**	0.5	49.6 (44.3–55.7)	–	>300	>6.0 (6 Hz)
**22**	0.5	>130	57.6 (33.9–97.9)	>300	>5.2 (6 Hz)
**24**	0.5	>130	43.8 (33.9–53.8)	>300	>6.8 (6 Hz)
**25**	0.5	87.9 (82.0–94.3)	88.9 (83.3–94.9)	210.5 (195.0–227.1)	2.4 (MES) 2.4 (6 Hz)
**27**	0.5	–	36.3 (24.9– 53.1)	>300	8.3 (6 Hz)
**28**	0.5	117.4 (108.4–127.3)	88.1 (81.8–95.0)	272.5 (253.2–293.3)	2.3 (MES) 2.9 (6 Hz)
**29**	0.5	55.4 (41.5–65.4)	49.3 (38.0–62.1)	178.8 (164.1–188.5)	3.2 (MES) 3.6 (6 Hz)
**30**	0.5	45.6 (35.8–58.2)	39.5 (28.3–55.2)	162.4 (144.0–183.1)	3.5 (MES) 4.1 (6 Hz)
**31**	0.5	–	103.8 (86.1–125.2)	>300	2.9 (6 Hz)
**LCS** *^f^*	0.5	9.2 (8.5–10.0)	5.3 (3.5–7.8)	46.2 (44.5–48.0)	5.0 (MES) 8.8 (6 Hz)
**LTG** *^f^*	1.0	3.1 (1.9–4.7)	6.4 (4.1–9.9)	48.3 (37.4–62.6)	15.6 (MES) 7.5 (6 Hz)
**VPA** *^f^*	0.5	252.7 (220.1–290.2)	130.6 (117.6–145.2)	430.7 (407.9–454.9)	1.7 (MES) 3.3 (6 Hz)

Values in parentheses are 95% confidence intervals determined by probit analysis [23]. ^*a*^ Time to peak effect; *^b^* ED_50_ (MES, maximal electroshock seizure test); *^c^* ED_50_ (6 Hz psychomotor seizure test, 32 mA); *^d^* TD_50_ (acute neurological toxicity determined in the rotarod test); *^e^* protective index (TD_50_/ED_50_); *^f^* reference antiepileptic drugs (AEDs): lacosamide (LCS), lamotrigine (LTG), and valproic acid (VPA) tested in the same conditions. TPEs for model AEDs taken from own experiments. A dash indicates not tested.

**Table 2:** In vitro binding assays for several compounds at concentration of 10 µM or 100 µM.

	% Inhibition of Control Specific Binding *^a^*				
Assay	Na^+^ Channel (Site 2) (Antagonist Radioligand) *^,#^	Cav_1.2_ Channel (Antagonist Radioligand) *	Cav_2.2_ Channel (Antagonist Radioligand) *	GABA-A Receptor (α_1_,β_2_,γ_2_) (Agonist Radioligand) *	Potassium Channel (hERG) **
**Concentration (µM)**	10	10	10	10	100
**Compound**					
**3**	10.8	9.0	3.6	5.2	0.8
**4**	23.5	5.0	1.1	2.8	2.3
**21**	18.8	26.6	0.2	1.2	6.3
**24**	22.3	**36.5**	5.5	−0.8	4.4
**25**	14.8	19.0	2.5	3.6	4.8
**27**	20.1	**38.3**	3.8	0.7	2.7
**29**	18.3	**44.2**	−0.6	2.1	3.4
**30**	15.8	**47.6**	4.0	0.3	1.7

The data for the most potent compounds have been bolded for better visualization. *^a^* Results showing activity higher than 50% are considered to represent a significant effect of the test compounds; results showing an inhibition between 25% and 50% (bolded values) are indicative of a moderate effect; results showing an inhibition lower than 25% are not considered significant. ^#^ Central nervous system (CNS) tetrodotoxin (TTX)-sensitive sodium channels that include Na_v_1.1, Na_v_1.2, Na_v_1.3, and Na_v_1.6 subtypes. Binding studies were performed commercially in Eurofins Cerep SA (Celle l’Evescault, France). Source: * rat cerebral cortex; ** human recombinant (HEK-293 cells).

**Table 3:** In vitro functional assays for several compounds at concentration of 10 µM.

Molecular Target	% Inhibition of Control Agonist Response *^a^*
Assay	Cav_1.2_ (*h*) Calcium Ion Channel Cell-Based Antagonist Calcium Flux Assay (Human Recombinant (HEK-293 Cells))
Compound	
**21**	27.0
**24**	**38.8**
**27**	**40.0**
**29**	**45.0**
**30**	**49.0**

The data for the most potent compounds have been bolded for better visualization. *^a^* Results showing an inhibition higher than 50% are considered to represent a significant effect of the test compounds; results showing an inhibition between 25% and 50% (bolded values) are indicative of a moderate effect; results showing an inhibition lower than 25% are not considered significant. Functional studies were performed commercially in Eurofins Panlabs Discovery Services Taiwan, Ltd. (New Taipei City, Taiwan).

**Table 4:** The results obtained in parallel artificial membrane permeability assay (PAMPA), expressed as the permeability coefficient (*Pe*).

Compound	*Pe* (10^−6^ cm/s) ± SD ^a^
CFN ^b^	12.22 ± 0.9
NFX ^c^	0.056 ± 0.0
**4**	11.25 ± 0.7
**24**	10.87 ± 0.5
**30**	13.20 ± 1.5

^a^ Tested in triplicate; ^b^ caffeine (CFN); ^c^ norfloxacin (NFX).

## Supplementary Materials

Supplementary Materials can be found at https://www.mdpi.com/1422-0067/21/22/8780/s1. General procedure for the preparation staring amine derivatives (A1 and A2); Table S1. Anticonvulsant activity and acute neurotoxicity screening data in the MES, 6 Hz (32 mA), and rotarod tests in mice i.p. (dose of 100 mg/kg)—compounds **3**–**13**; Table S2. Anticonvulsant activity and acute neurotoxicity screening data in the MES, 6 Hz (32 mA), and rotarod tests in mice i.p. (dose of 100 mg/kg)—compounds **14**–**24**; Table S3. Anticonvulsant activity and acute neurotoxicity screening data in the MES, 6 Hz (32 mA), and rotarod tests in mice i.p. (dose of 100 mg/kg)—compounds **25**–**31**; Table S4. In vitro binding and functional assays; Figure S1. UPLC chromatogram after 120 min incubation of compound 4 with HLMs; Figure S2. MS ion fragment analyses and most probable structures of compound **4** metabolites M1–M3; Figure S3. UPLC chromatogram after 120 min incubation of compound **24** with HLMs; Figure S4. MS ion fragment analyses and most probable structure of 24 metabolite M1; Figure S5. UPLC chromatogram after 120 min incubation of compound **30** with HLMs; Figure S6. MS ion fragment analyses and most probable structures of 30 metabolites M1 and M2; Figure S7. The MetaSite 6.0.1. software predictions of the most probably sites of compounds **4**, **24**, and **30** metabolism; ^1^H-NMR and ^13^C-NMR spectra for selected final compounds.

## Abbreviations

ADME-Tox	Absorption, distribution, metabolism, excretion, toxicity
AEDs	Antiepileptic drugs
CCCP	3-Chlorophenylhydrazone
CDI	Carbonyldiimidazole
CFN	Caffeine
CNS	Central nervous system
DCM	Dichloromethane
DMF	Dimethylformamide
DX	Doxorubicin
HLMs	Human liver microsomes
HMDS	Hexamethyldisilazane
6 Hz	6 Hz seizure test
KE	Ketoconazole
LCS	Lacosamide
LiAlH_4_	Lithium aluminum hydride
LTG	Lamotrigine
Lacosamide	Acetonitrile
MES	Maximal electroshock seizure test
MeOH	Methanol
NFX	Norfloxacin
OXPT	Oxaliplatin
PAMPA	Parallel artificial membrane permeability assay
PI	Protective index (TD_50_/ED_50_)
QD	Quinidine
SE	Sulfaphenazole
THF	Tetrahydrofuran
TPE	Time of peak effect
VPA	Valproic acid
