# Supplementary material for: The Search for New Anticonvulsants in a Group of (2,5-Dioxopyrrolidin-1-yl)(phenyl)Acetamides with Hybrid Structure—Synthesis and In Vivo/In Vitro Studies
Source: Int J Mol Sci. 2020 Nov 20;21(22):8780. doi: 10.3390/ijms21228780 (PMC7699745; doi:10.3390/ijms21228780)

**Supplementary Materials**

# The search for new anticonvulsants in a group of (2,5-dioxopyrrolidin-1-yl)(phenyl)acetamides with hybrid structure - synthesis and *in vivo*/*in vitro* studies

**Michał Abram [1], Marcin Jakubiec [1], Anna Rapacz [2], Szczepan Mogilski [2], Gniewomir Latacz [3], Rafał M. Kamiński [1], Krzysztof Kamiński [1,*]**

[1] Jagiellonian University Medical College, Faculty of Pharmacy, Department of Medicinal Chemistry, Medyczna 9, 30-688 Cracow, Poland; k.kaminski@uj.edu.pl (K.K.); michal.abram@doctoral.uj.edu.pl (M.A.); marcin.jakubiec@doctoral.uj.edu.pl (M.J.); rafa1.kaminski@uj.edu.pl (R.M.K.)
[2] Jagiellonian University Medical College, Faculty of Pharmacy, Department of Pharmacodynamics, Medyczna 9, 30-688 Cracow, Poland; a.rapacz@uj.edu.pl (A.R.); szczepan.mogilski@uj.edu.pl (S.M.)
[3] Jagiellonian University Medical College, Faculty of Pharmacy, Department of Technology and Biotechnology of Drugs, Medyczna 9, 30-688 Cracow, Poland; gniewomir.latacz@uj.edu.pl (G.L.)
* Correspondence: k.kaminski@uj.edu.pl; Tel.: + 48 12 620 54 59

## Table of contents

General procedure for the preparation staring amine derivatives (**A1** and **A2**) .................................. 2

**Table S1**. Anticonvulsant activity and acute neurotoxicity screening data in the MES, 6 Hz (32 mA), and rotarod tests in mice *i.p.* (dose of 100 mg/kg) – compounds **3–13**...................................................... 3

**Table S2**. Anticonvulsant activity and acute neurotoxicity screening data in the MES, 6 Hz (32 mA), and rotarod tests in mice *i.p.* (dose of 100 mg/kg) – compounds **14–24**. .................................................... 4

**Table S3**. Anticonvulsant activity and acute neurotoxicity screening data in the MES, 6 Hz (32 mA), and rotarod tests in mice *i.p.* (dose of 100 mg/kg) – compounds **25–31**. .................................................... 5

**Table S4**. *In vitro* binding and functional assays. ............................................................................ 6

**Figure S1**. UPLC chromatogram after 120 min incubation of compound **4** with HLMs....................... 7

**Figure S2**. MS ion fragment analyses and most probable structures of compound **4** metabolites **M1**-**M3**. ......................................................................................................................................... 8

**Figure S3**. UPLC chromatogram after 120 min incubation of compound **24** with HLMs..................... 9

**Figure S4**. MS ion fragment analyses and most probable structure of **24** metabolite **M1**. .................. 10

**Figure S5**. UPLC chromatogram after 120 min incubation of compound **30** with HLMs................... 11

**Figure S6**. MS ion fragment analyses and most probable structures of **30** metabolites **M1** and **M2**. ... 12

**Figure S7**. The MetaSite 6.0.1. software predictions of the most probably sites of compounds **4**, **24**, and **30** metabolism. ........................................................................................................ 13

$^{1}$H NMR, $^{13}$C NMR spectra for selected final compounds ............................................................ 14

## Supplementary Materials

## General procedure for the preparation staring amine derivatives (A1 and A2)

The synthetic pathway for amines **A1** and **A2** is shown in Scheme S1.

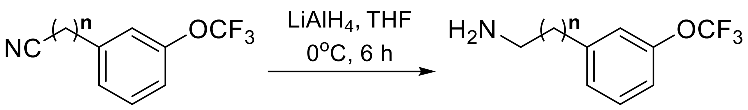


**A1** (n = 0)
**A2** (n = 1)

**Scheme S1.** Synthesis of starting amine derivatives **A1 and A2**.

A solution of the given nitrile derivative (1 g) in anhydrous THF (20 mL) was added dropwise to a suspension of $LiAlH_4$ (3 equiv) in anhydrous THF (20 mL) at 0°C. Next, the mixture was stirred at room temperature for 6 h. The progress of the reaction was monitored by HPLC. After the reaction was completed, water (10 mL) was added dropwise at 0°C followed by an aqueous 10% NaOH solution (10 mL). The reaction mixture was stirred at room temperature (0.5 h), and then was filtered through Celite 545 (Merck, Darmstadt, Germany). Next, the obtained solution was extracted with DCM (3 × 20 ml). The organic layer was dried over anhydrous $Na_2SO_4$, and then the solvents were evaporated under reduced pressure to obtain the desired amines as brown oils. Starting amines **A1** and **A2** were advanced to further reactions without purification.

*(3-(trifluoromethoxy)phenyl)methanamine* (**A1**)
Brown oil, Yield: 71.4%, (0.73 g); TLC: $R_f$ = 0.23 ($S_1$); UPLC (purity 85.9%): $t_R$ = 2.85 min. LC-MS (ESI): *m/z* calcd for $C_8H_8F_3NO$ $(M+H)^+$ 192.06, found 192.0.

*2-(3-(trifluoromethoxy)phenyl)ethan-1-amine* (**A2**)
Brown oil, Yield: 68.2%, (0.7 g); TLC: $R_f$ = 0.28 ($S_1$); UPLC (purity 82.3%): $t_R$ = 3.42 min. LC-MS (ESI): *m/z* calcd for $C_9H_{10}F_3NO$ $(M+H)^+$ 206.07, found 206.2.

## Supplementary Materials

**Table S1.** Anticonvulsant activity and acute neurotoxicity screening data in the MES, 6 Hz (32 mA), and rotarod tests in mice *i.p.* (dose of 100 mg/kg) – compounds **3–13**.

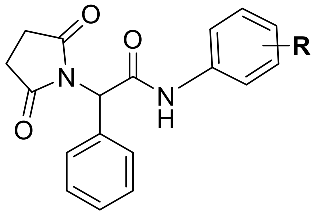


**3-13**

| **Compd** | **R** | **MES test** | **6 Hz (32 mA) test** | **Rotarod test** |
|---|---|---|---|---|
| **3** | H | **2/4** | **3/4** | 0/4 |
| **4** | 2-F | **4/4** | **1/4** | 0/4 |
| **5** | 3-F | 0/4 | **1/4** | - |
| **6** | 4-F | **2/4** | **2/4** | 0/4 |
| **7** | 2-Cl | 0/4 | **1/4** | - |
| **8** | 3-Cl | **3/4** | **1/4** | 0/4 |
| **9** | 4-Cl | **3/4** | **1/4** | 0/4 |
| **10** | 2-$CF_3$ | 0/4 | - | - |
| **11** | 3-$CF_3$ | 1/4 | - | - |
| **12** | 4-$CF_3$ | 0/4 | - | - |
| **13** | 3-$OCF_3$ | 0/4 | - | - |

Ratios where at least 50% of animals were protected have been highlighted in blue for easier data interpretation.
[a] Data indicate: number of mice protected / number of mice tested. The animals were examined 0.5 h after *i.p.* administration of compound. A dash indicates-not tested.

## Supplementary Materials

**Table S2.** Anticonvulsant activity and acute neurotoxicity screening data in the MES, 6 Hz (32 mA), and rotarod tests in mice *i.p.* (dose of 100 mg/kg) – compounds **14–24**.

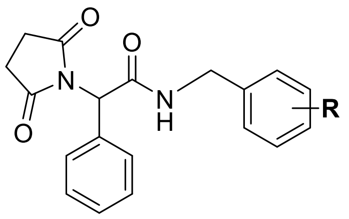

**14-24**

| Compd | R | MES test | 6 Hz (32 mA) test | Rotarod test |
|---|---|---|---|---|
| **14** | H | **2/4** | **2/4** | 1/4 |
| **15** | 2-F | 1/4 | - | - |
| **16** | 3-F | 1/4 | - | - |
| **17** | 4-F | 0/4 | - | - |
| **18** | 2-Cl | 0/4 | - | - |
| **19** | 3-Cl | **3/4** | **2/4** | 0/4 |
| **20** | 4-Cl | 0/4 | - | - |
| **21** | 2-$CF_3$ | **2/4** | 1/4 | 0/4 |
| **22** | 3-$CF_3$ | **3/4** | **2/4** | 0/4 |
| **23** | 4-$CF_3$ | 0/4 | - | - |
| **24** | 3-$OCF_3$ | **3/4** | **4/4** | 0/4 |

Ratios where at least 50% of animals were protected have been highlighted in blue for easier data interpretation.
[a] Data indicate: number of mice protected / number of mice tested. The animals were examined 0.5 h after *i.p.* administration of compound. A dash indicates-not tested.

## Supplementary Materials

**Table S3.** Anticonvulsant activity and acute neurotoxicity screening data in the MES, 6 Hz (32 mA), and rotarod tests in mice *i.p.* (dose of 100 mg/kg) – compounds **25–31**.

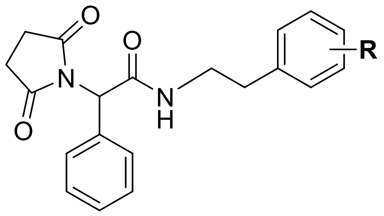

**25-30**

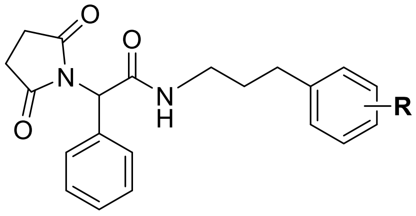

**31**

| Compd | R | MES test | 6 Hz (32 mA) test | Rotarod test |
|---|---|---|---|---|
| **25** | H | **3/4** | **3/4** | 1/4 |
| **26** | 2-F | **1/4** | - | - |
| **27** | 3-Cl | **2/4** | **3/4** | 0/4 |
| **28** | 4-Cl | **4/4** | **3/4** | 1/4 |
| **29** | 3-$CF_3$ | **3/4** | **3/4** | 0/4 |
| **30** | 3-$OCF_3$ | **4/4** | **3/4** | 0/4 |
| **31** | H | **2/4** | **2/4** | 0/4 |

Ratios where at least 50% of animals were protected have been highlighted in blue for easier data interpretation.
[a] Data indicate: number of mice protected / number of mice tested. The animals were examined 0.5 h after *i.p.* administration of compound. A dash indicates-not tested.

## Supplementary Materials

Binding studies were carried out commercially in Eurofines Laboratories (Poitiers, France). The functional assays were performed in Eurofins Panlabs Discovery Services Taiwan, Ltd. (New Taipei City, Taiwan). All procedures are described elsewhere (**Table S4**).

**Table S4.** *In vitro* binding and functional assays.

| **Binding studies** | Ref. |
|---|---|
| $Na^+$ channel (site 2) | 6 |
| $Cav_{1.2}$ channel (antagonist radioligand) | 7 |
| $Cav_{2.2}$ channel (antagonist radioligand) | 12 |
| GABA-A receptor ($\alpha_1$,$\beta_2$,$\gamma_2$) (agonist radioligand) | 13 |
| Potassium channel (hERG) | 14 |
| **Functional studies** | |
| $Cav_{1.2}$ *(h)* calcium ion channel cell based antagonist calcium flux assay | 16,17 |

## Supplementary Materials

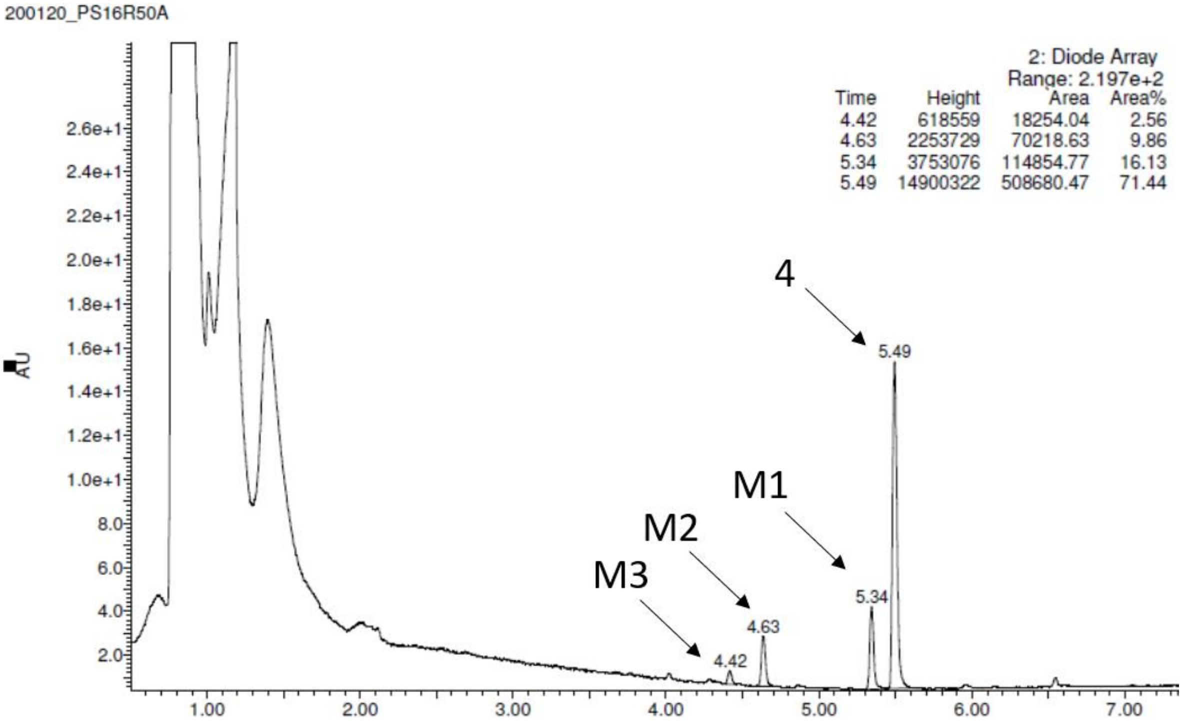


**Figure S1**. UPLC chromatogram after 120 min incubation of compound **4** with HLMs. 71.44% of parent compound remained in the reaction mixture. Three metabolites (**M1**, m/z = 359.21; **M2**, m/z = 345.21; **M3**, m/z = 343.12) were determined.

## Supplementary Materials

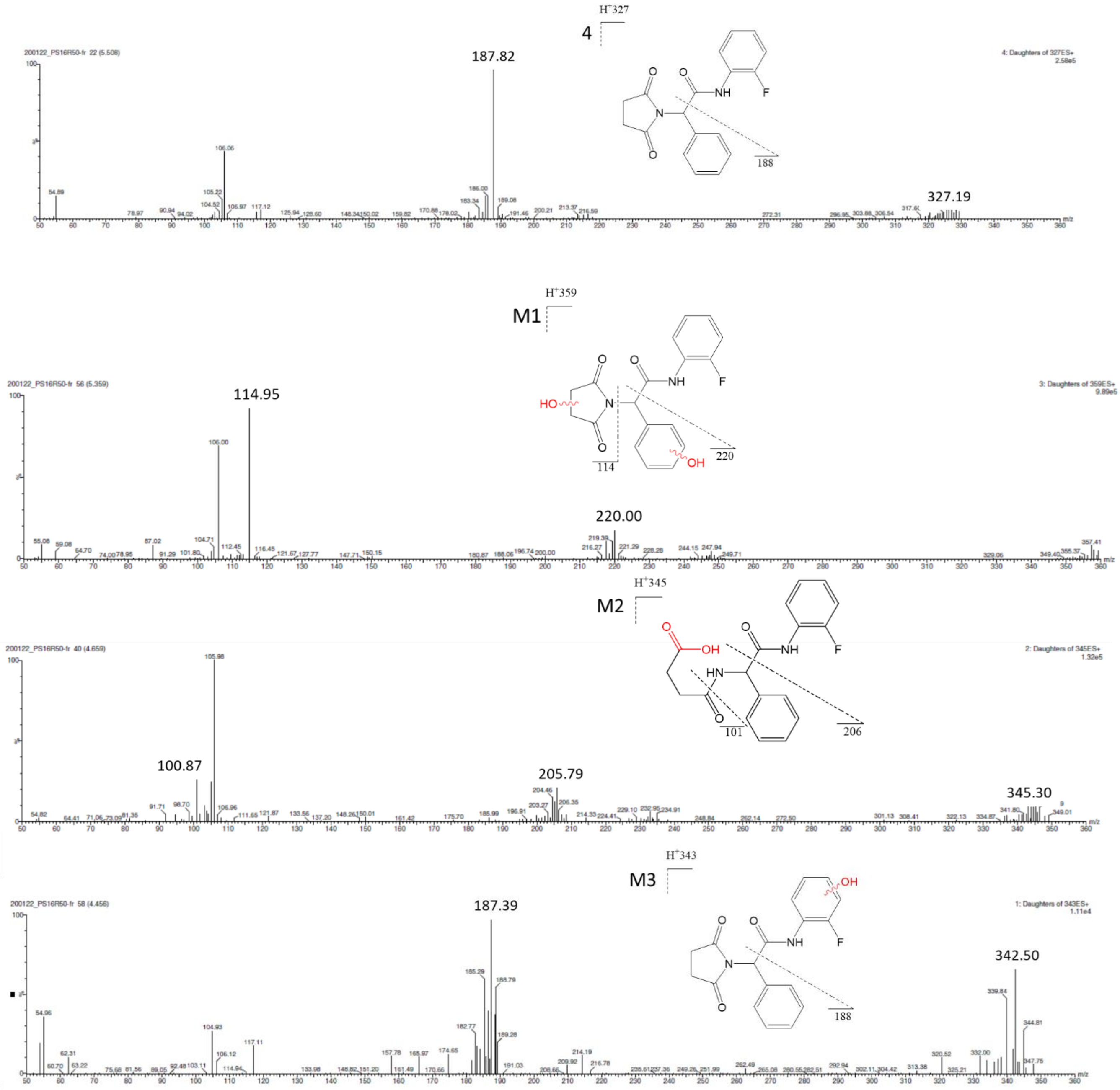


**Figure S2**. MS ion fragment analyses and most probable structures of compound **4** metabolites **M1**-**M3**.

## Supplementary Materials

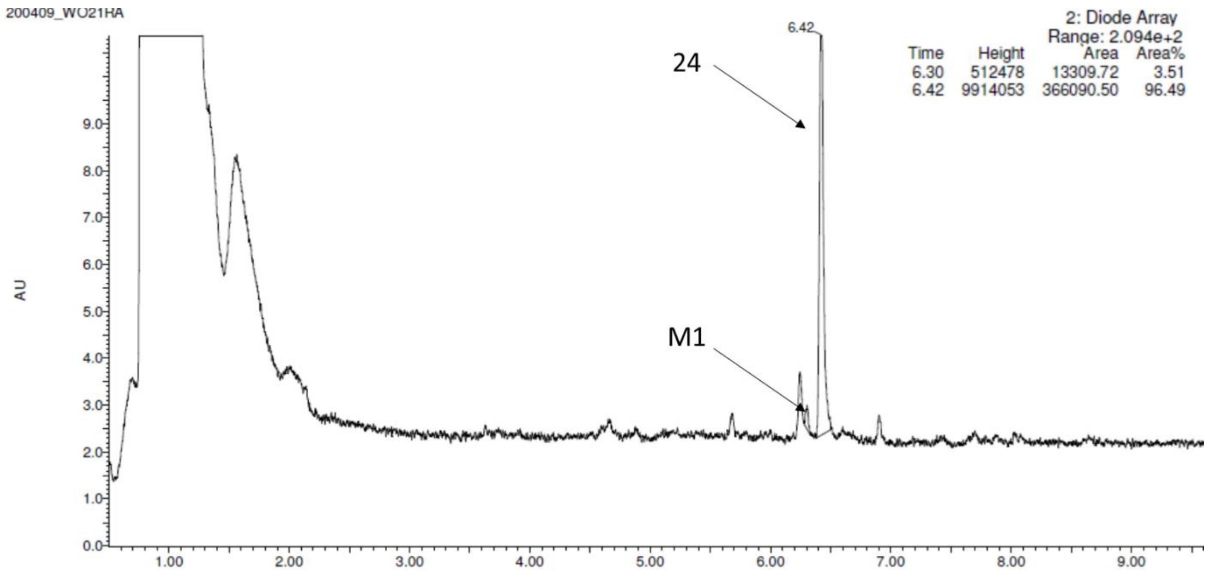


**Figure S3**. UPLC chromatogram after 120 min incubation of compound **24** with HLMs. 96.49% of parent compound remained in the reaction mixture. One metabolite (**M1**, m/z = 439.2) was determined.

## Supplementary Materials

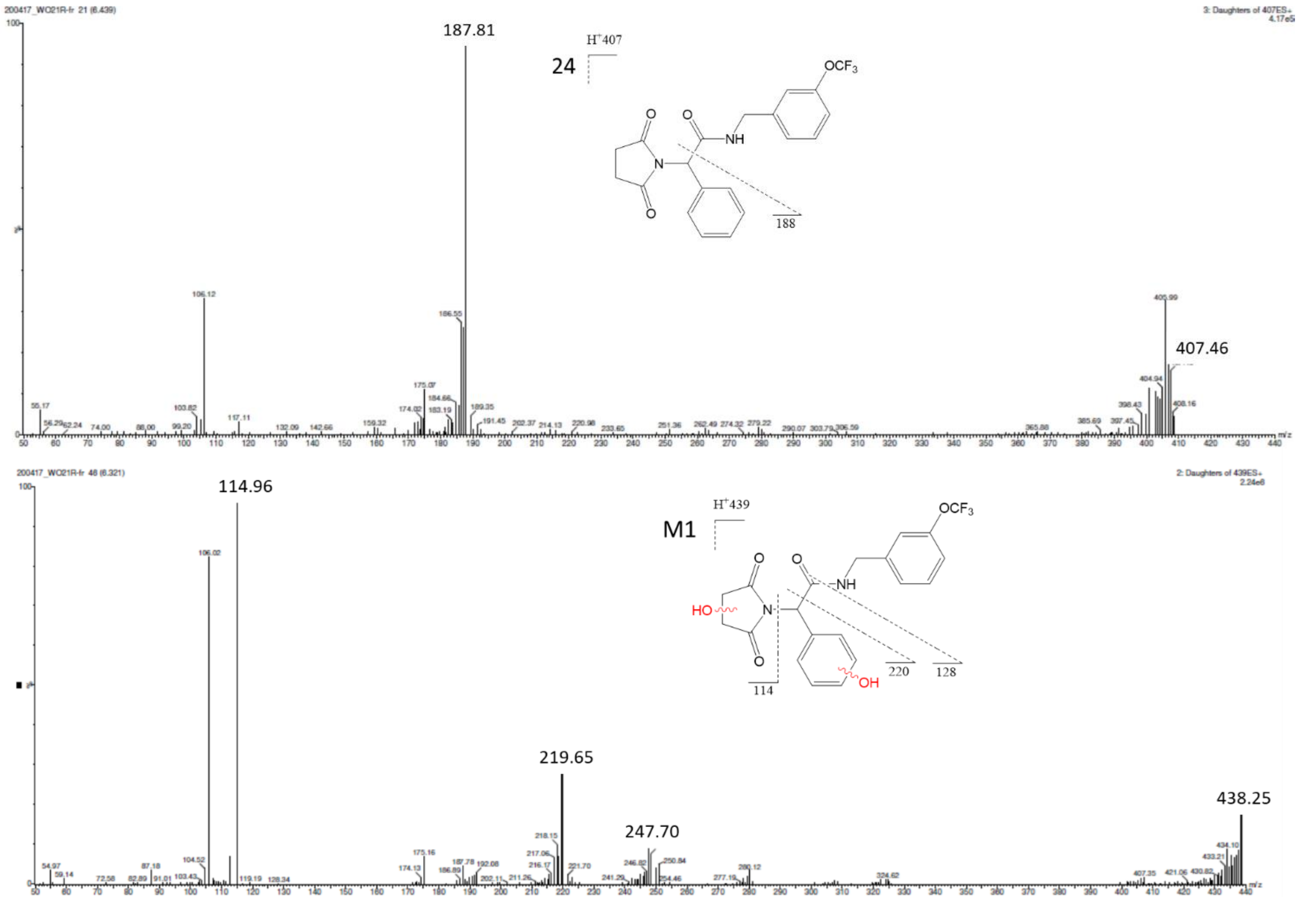


**Figure S4**. MS ion fragment analyses and most probable structure of **24** metabolite **M1**.

## Supplementary Materials

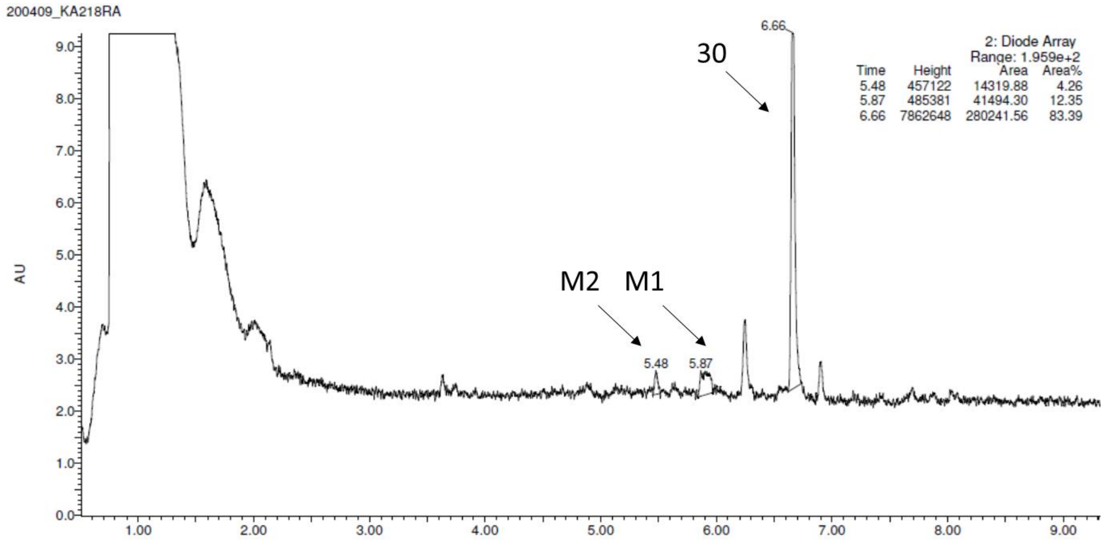


**Figure S5**. UPLC chromatogram after 120 min incubation of compound **30** with HLMs**.** 83.39% of parent compound remained in the reaction mixture. Two metabolites (**M1**, m/z = 437.2; **M2**, m/z = 437.2) were determined.

**Supplementary Materials**

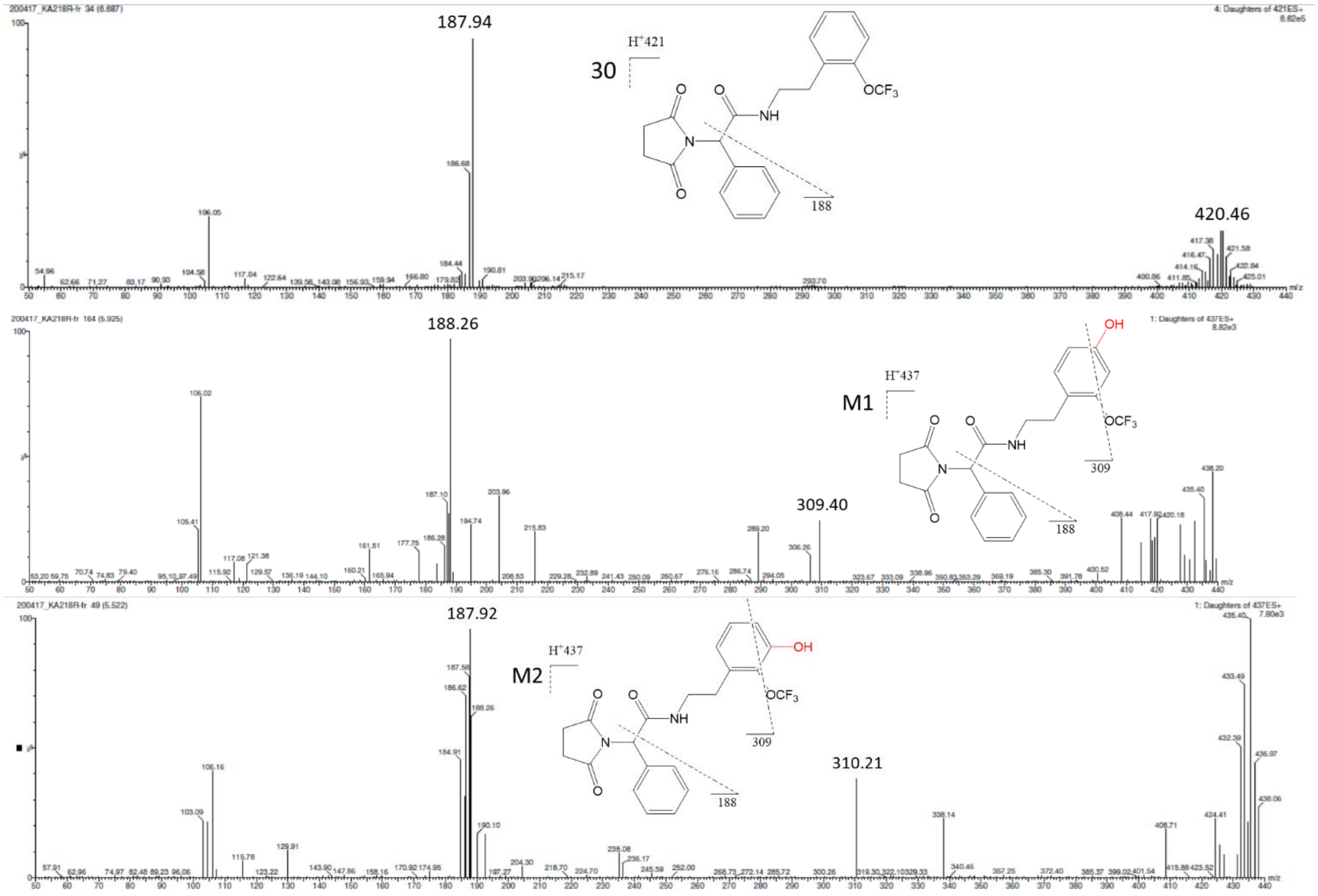


**Figure S6**. MS ion fragment analyses and most probable structures of **30** metabolites **M1** and **M2**.

## Supplementary Materials

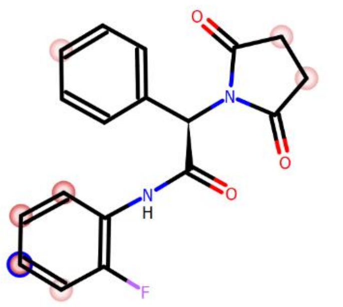


**4**

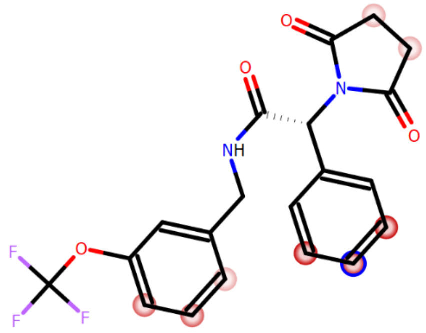


**24**

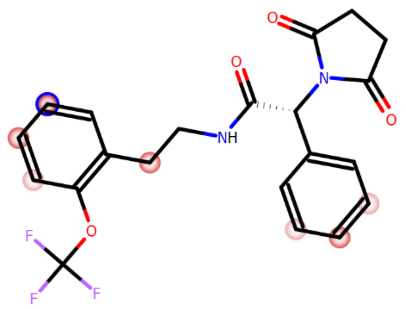


**30**

**Figure S7**. The MetaSite 6.0.1. software predictions of the most probably sites of compounds **4**, **24**, and **30** metabolism. The darker red color - the higher probability to be involved in the metabolism pathway. The blue circle marked the site of compound with the highest probability of metabolic bioconversion.

## Supplementary Materials

## $^{1}$H NMR, $^{13}$C NMR spectra for selected final compounds

*2-(2,5-Dioxopyrrolidin-1-yl)-N,2-diphenylacetamide* (**3**)- $^{1}$H NMR

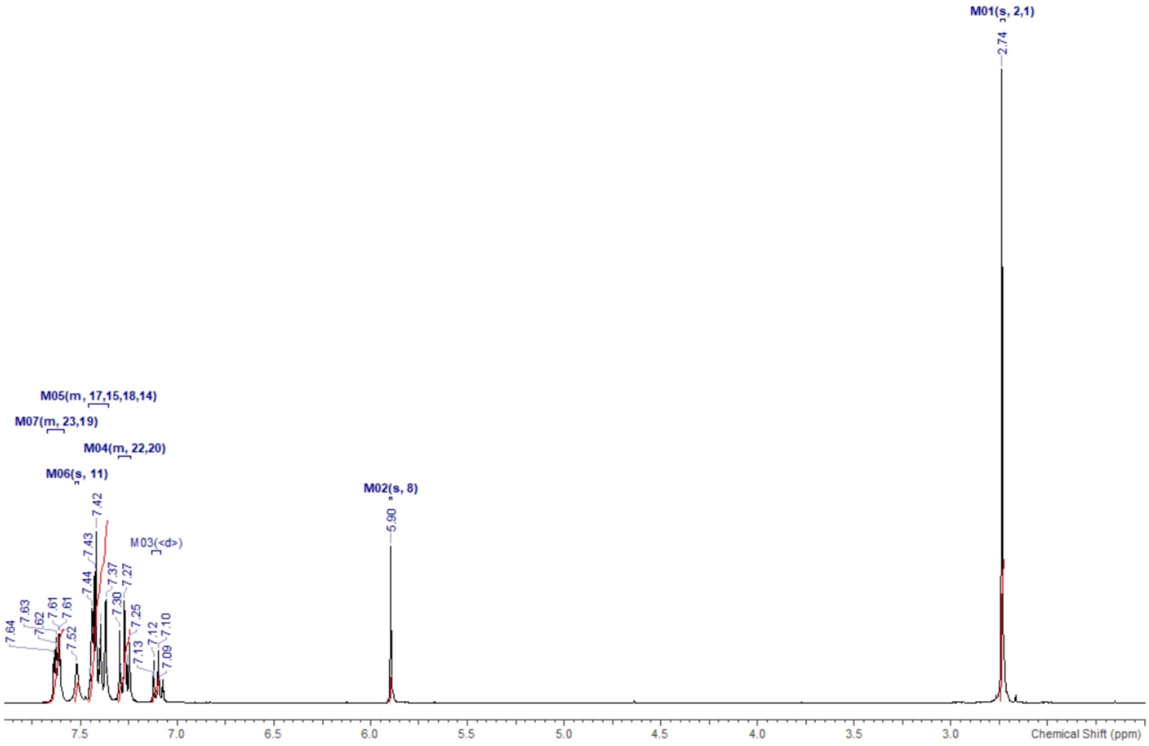


*2-(2,5-Dioxopyrrolidin-1-yl)-N,2-diphenylacetamide* (**3**)- $^{13}$C NMR

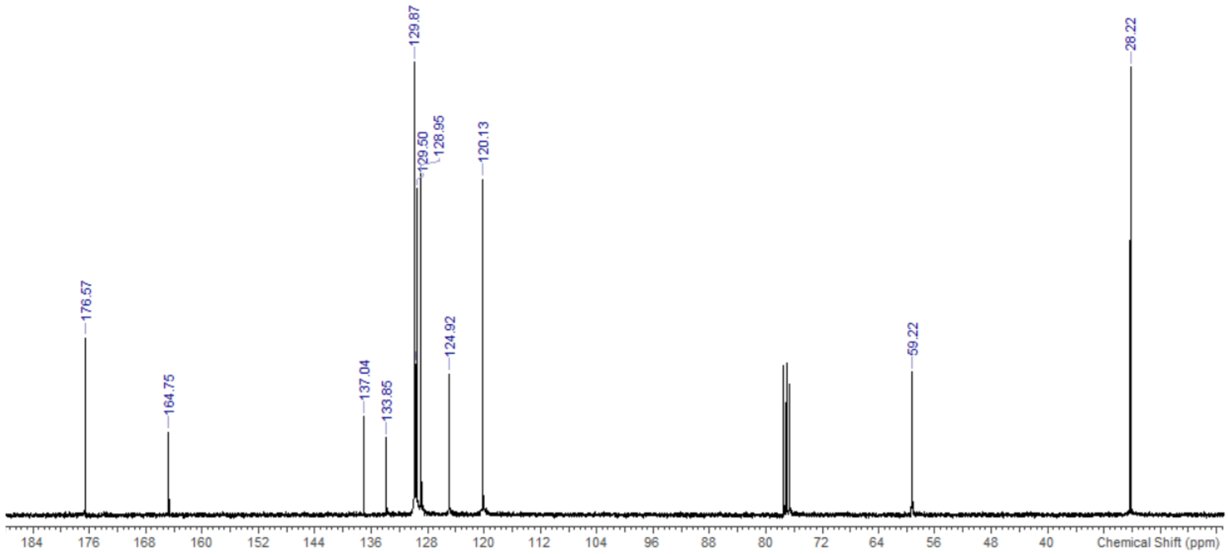

## Supplementary Materials

*2-(2,5-Dioxopyrrolidin-1-yl)-N-(2-fluorophenyl)-2-phenylacetamide* (**4**)- $^1$H NMR

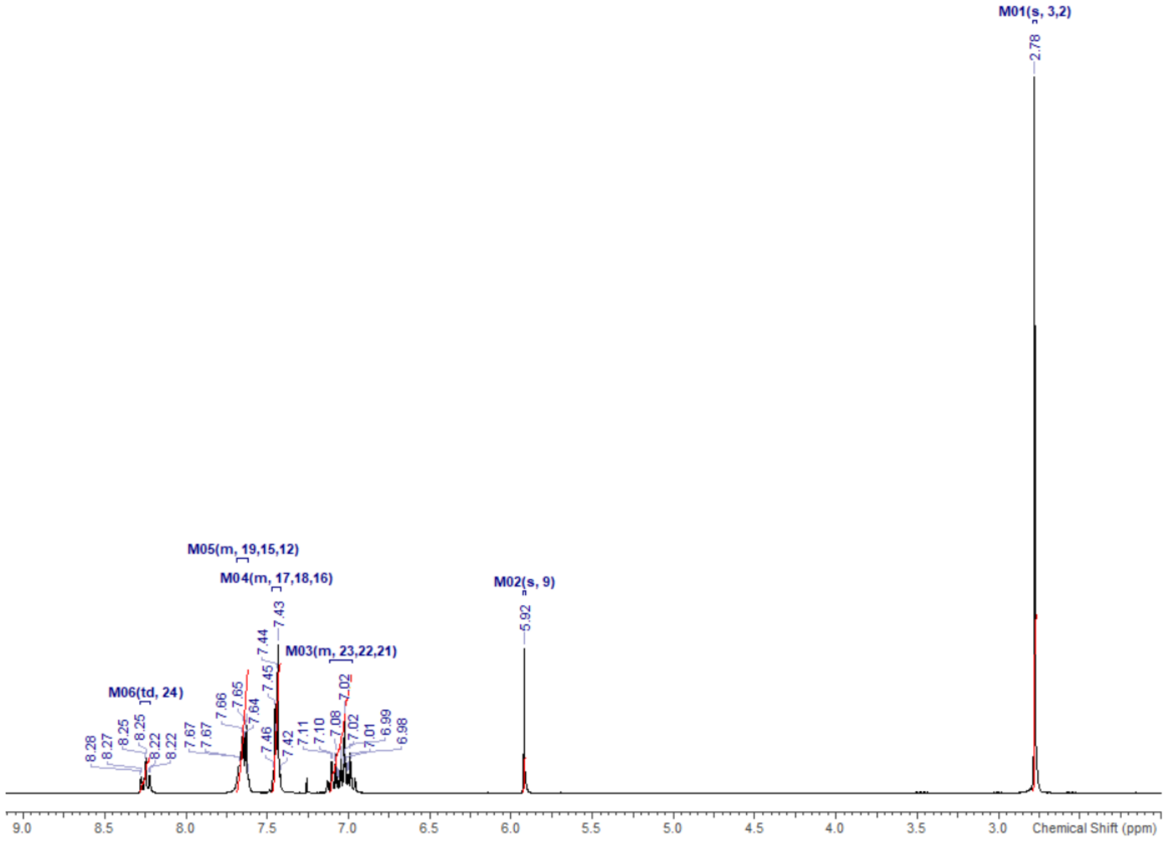


*2-(2,5-Dioxopyrrolidin-1-yl)-N-(2-fluorophenyl)-2-phenylacetamide* (**4**)- $^1$H NMR

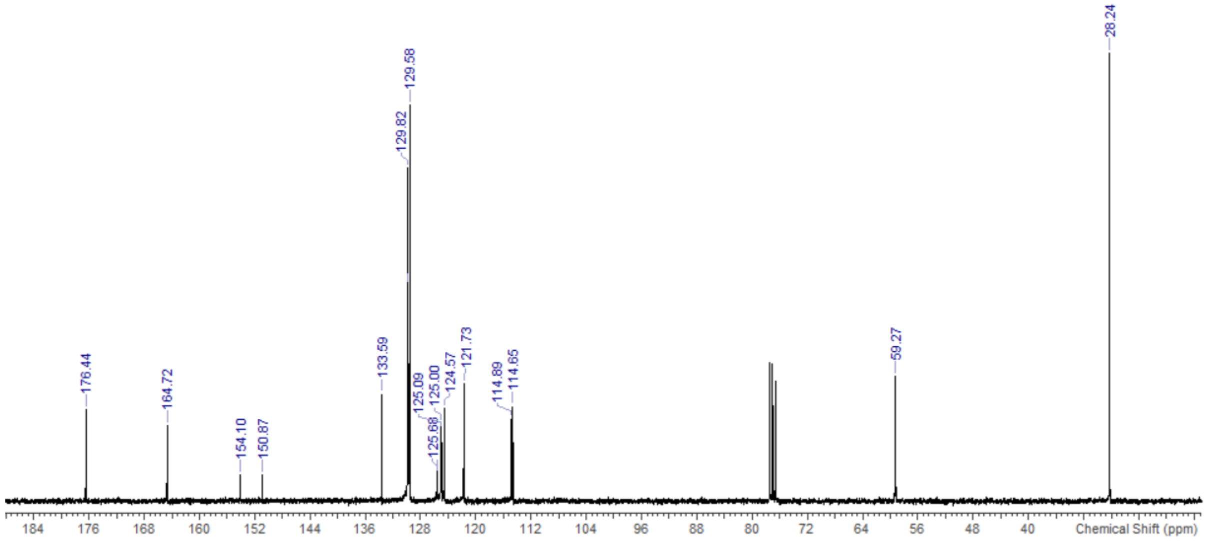

## Supplementary Materials

*2-(2,5-Dioxopyrrolidin-1-yl)-N-(2-fluorobenzyl)-2-phenylacetamide* (**15**)- $^{1}$H NMR (W0-2)

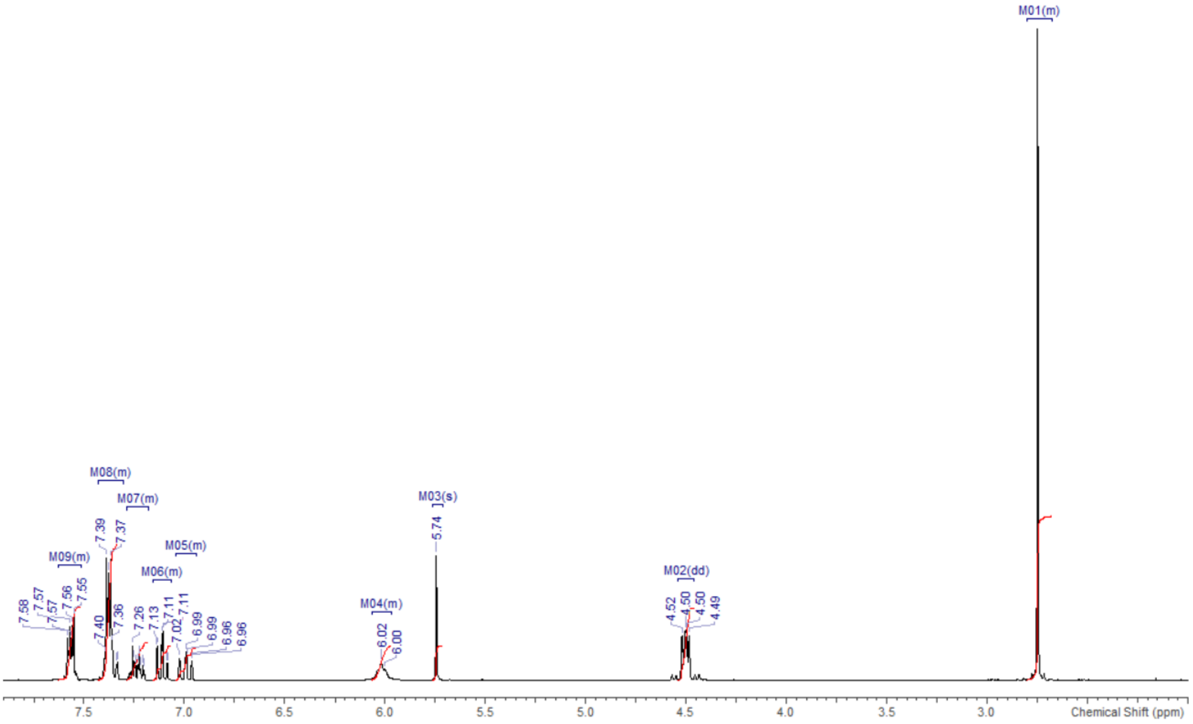


*2-(2,5-Dioxopyrrolidin-1-yl)-N-(2-fluorobenzyl)-2-phenylacetamide* (**15**)- $^{13}$C NMR (W0-2)

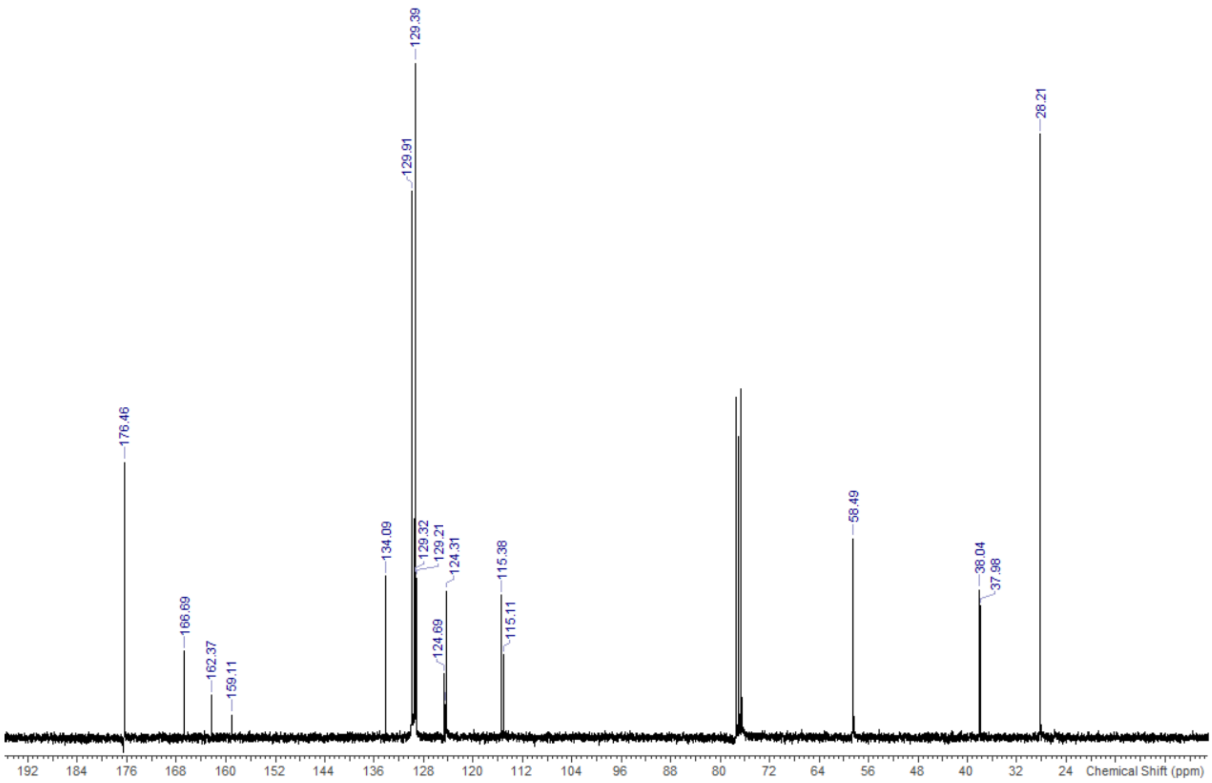

## Supplementary Materials

*2-(2,5-Dioxopyrrolidin-1-yl)-2-phenyl-N-(3-(trifluoromethyl)benzyl)acetamide* (**22**) – $^{1}$H NMR

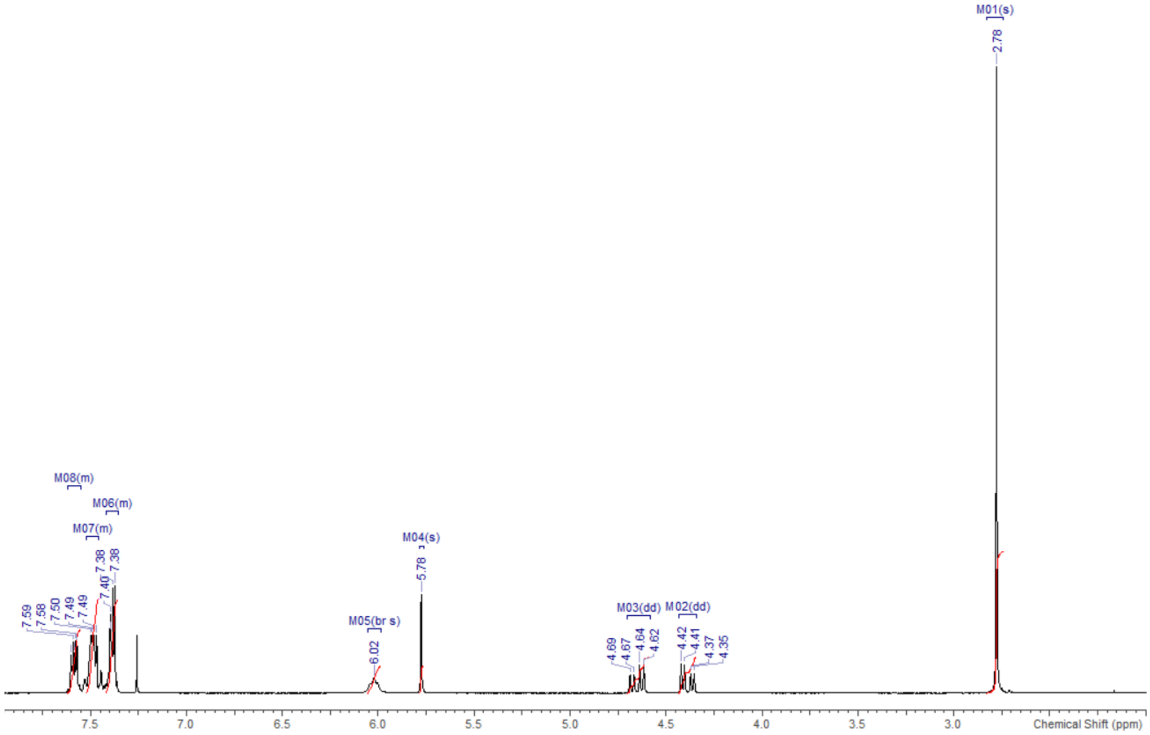

*2-(2,5-Dioxopyrrolidin-1-yl)-2-phenyl-N-(3-(trifluoromethyl)benzyl)acetamide* (**22**) - $^{13}$C NMR

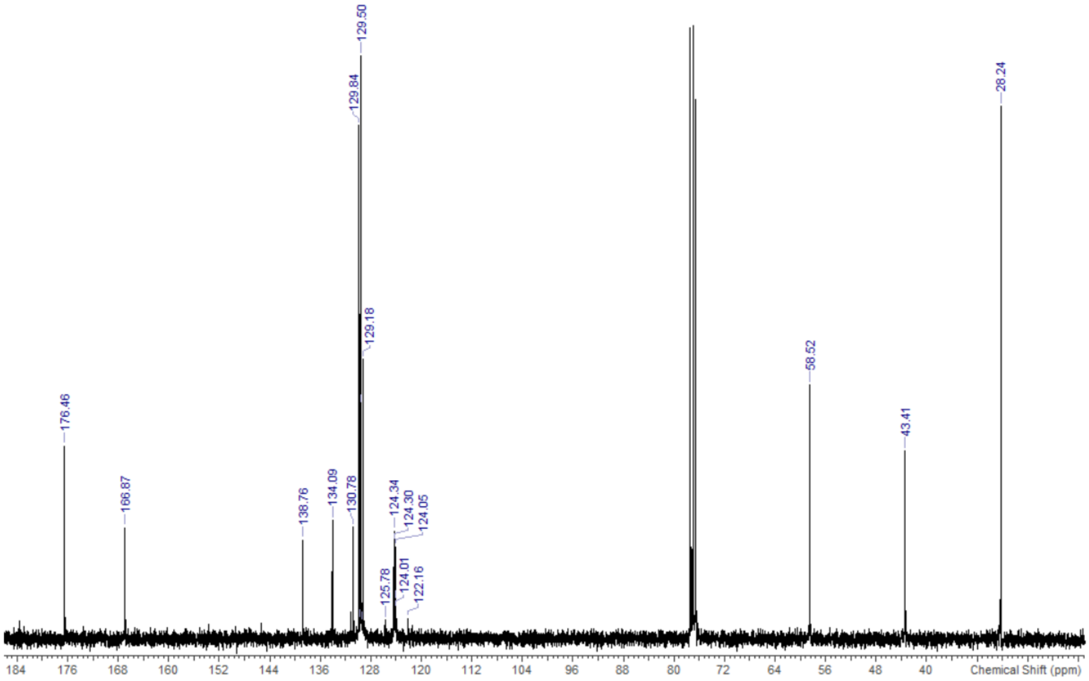

## Supplementary Materials

*2-(2,5-Dioxopyrrolidin-1-yl)-N-phenethyl-2-phenylacetamide* (**25**) $^{1}$H NMR

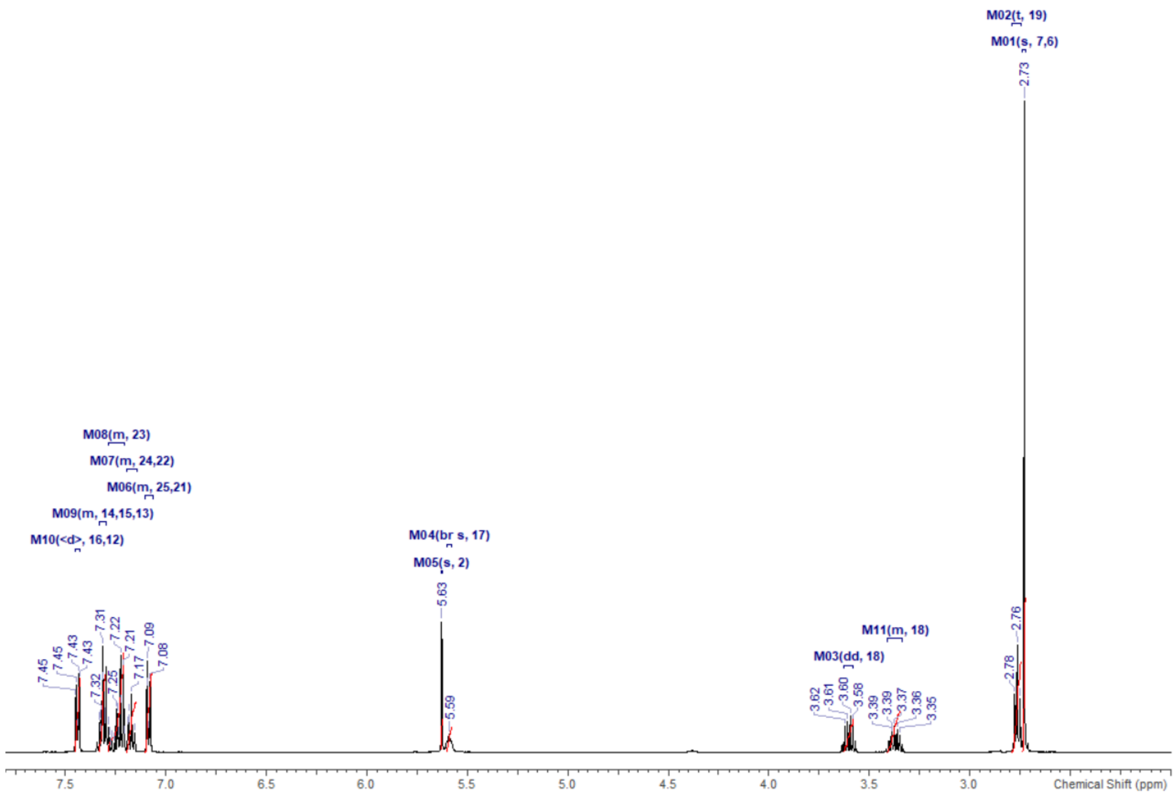


*2-(2,5-Dioxopyrrolidin-1-yl)-N-phenethyl-2-phenylacetamide* (**25**) $^{13}$C NMR

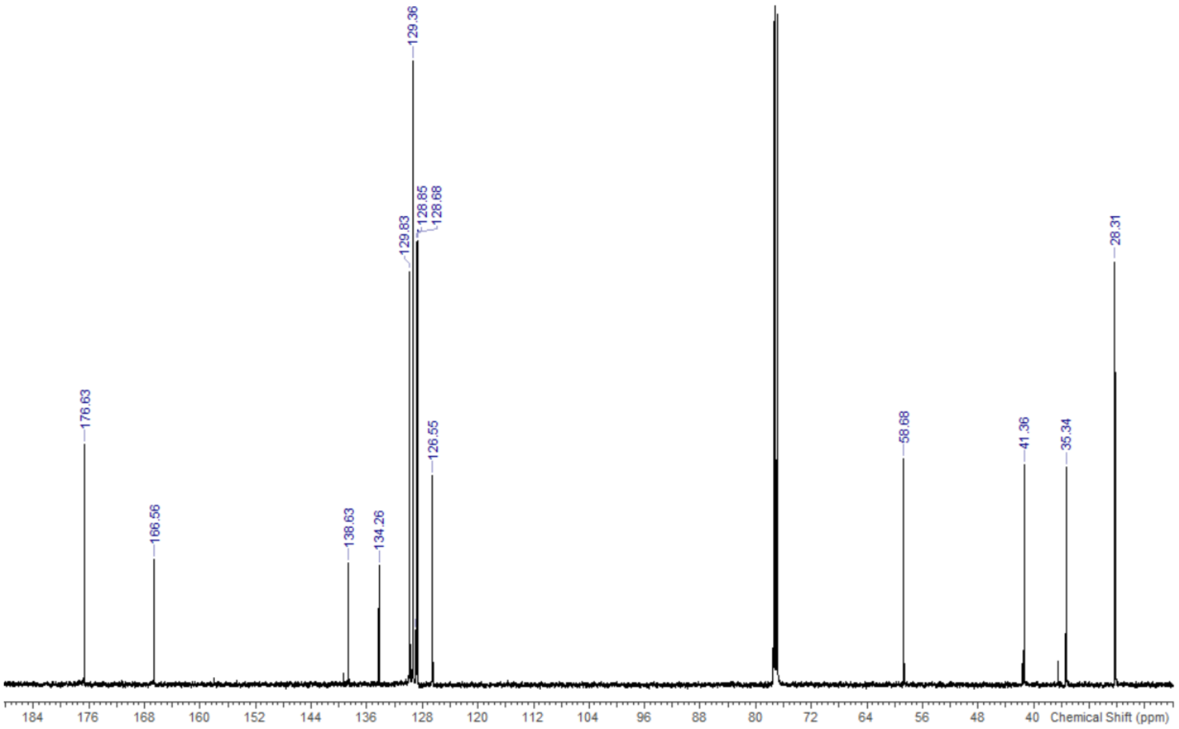

## Supplementary Materials

*2-(2,5-Dioxopyrrolidin-1-yl)-2-phenyl-N-(3-(trifluoromethoxy)phenethyl)acetamide* (**30**)- $^{1}$H NMR

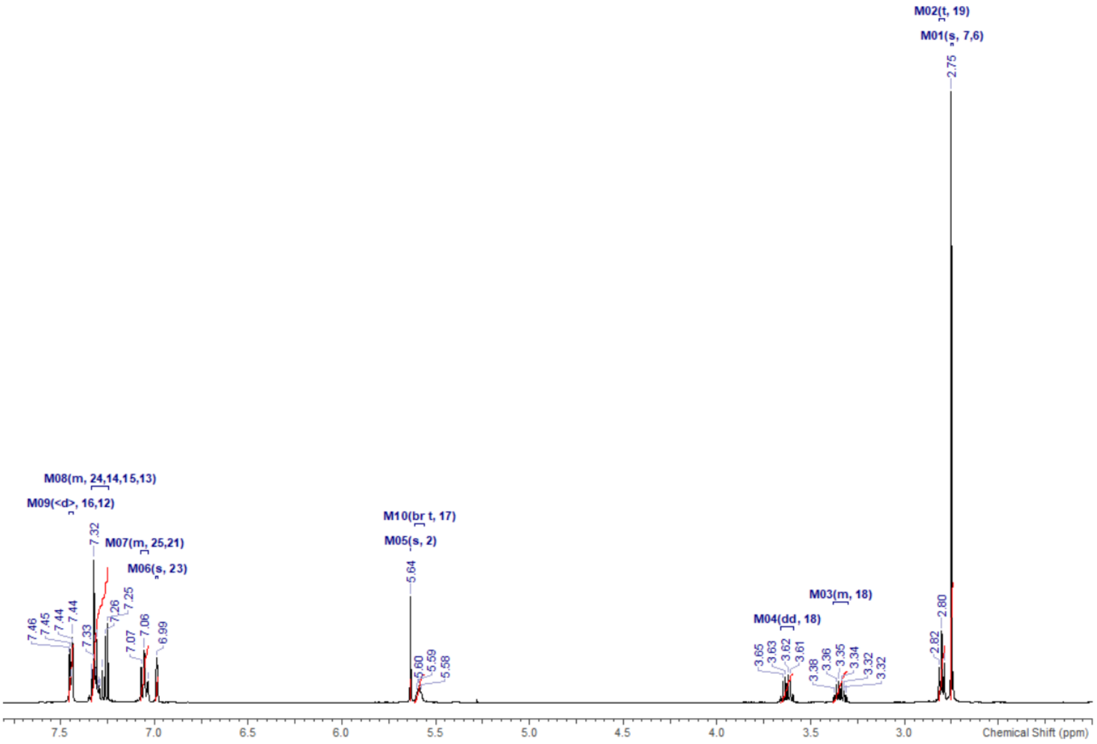


*2-(2,5-Dioxopyrrolidin-1-yl)-2-phenyl-N-(3-(trifluoromethoxy)phenethyl)acetamide* (**30**)- $^{13}$C NMR

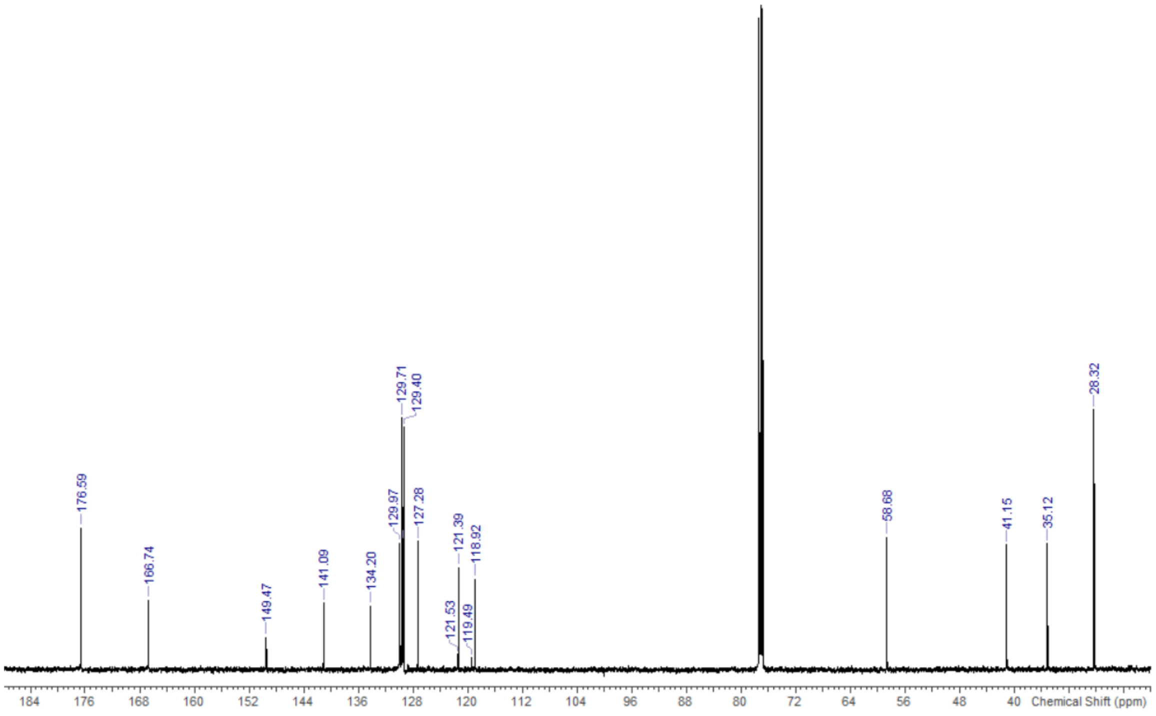

## Supplementary Materials

*2-(2,5-Dioxopyrrolidin-1-yl)-2-phenyl-N-(3-phenylpropyl)acetamide* (**31**) $^{1}$H NMR

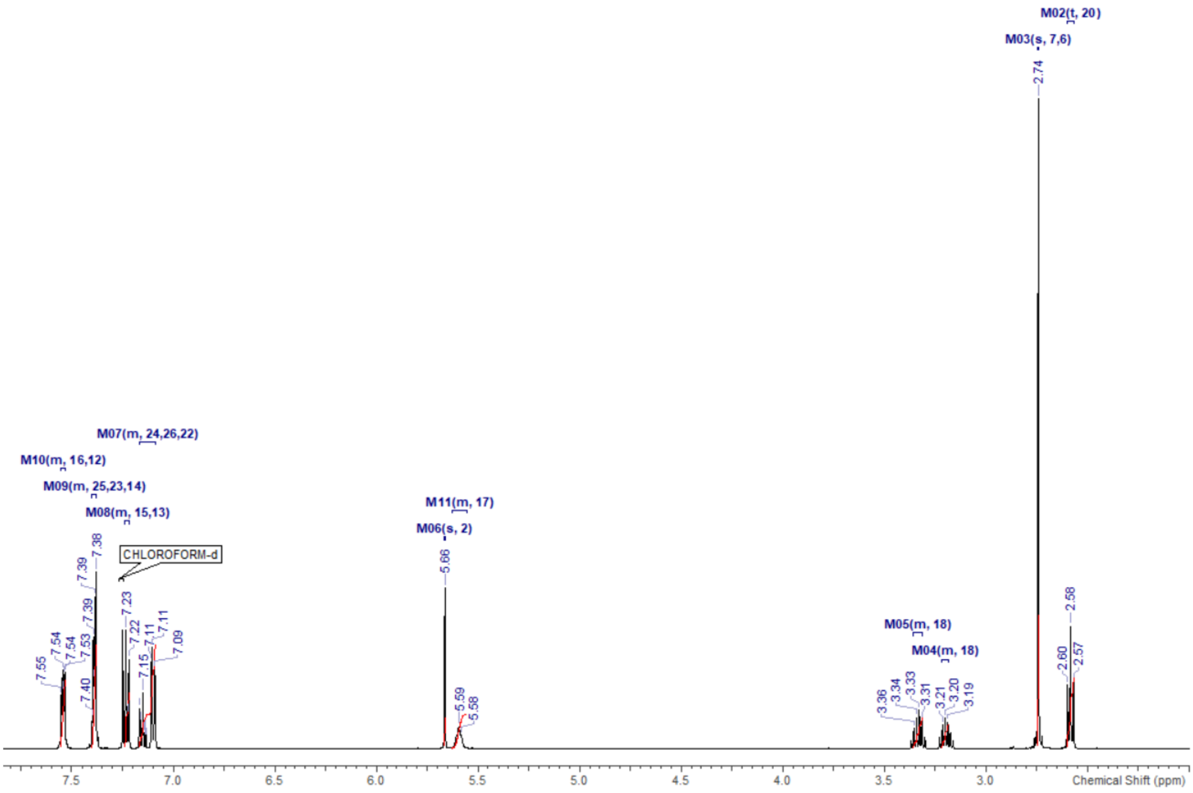


*2-(2,5-Dioxopyrrolidin-1-yl)-2-phenyl-N-(3-phenylpropyl)acetamide* (**31**) $^{13}$C NMR

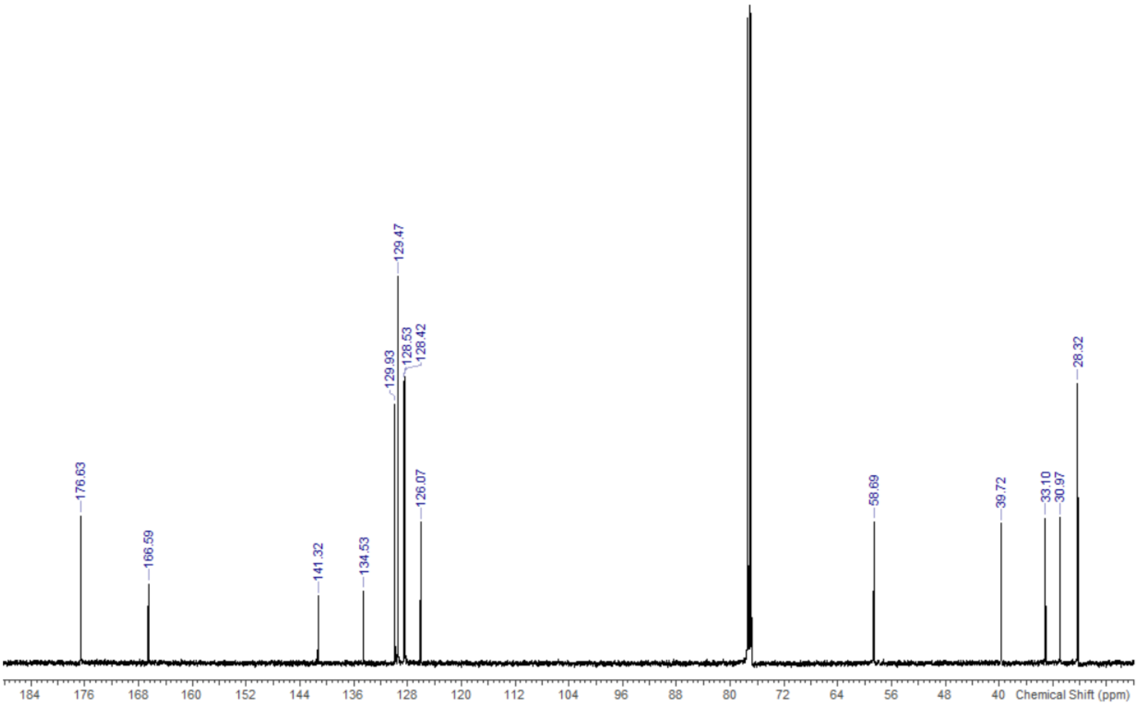

Supplement: Supplementary file 1 [file ijms-21-08780-s001.pdf]
